# Oxford Nanopore Technologies R10 sequencing enables accurate cgMLST-based bacterial outbreak investigation of *Neisseria meningitidis* and *Salmonella enterica* when accounting for methylation-related errors

**DOI:** 10.1128/jcm.00410-25

**Published:** 2025-08-22

**Authors:** Bert Bogaerts, Margo Maex, Florian Commans, Nathalie Goeders, An Van den Bossche, Sigrid C. J. De Keersmaecker, Nancy H. C. Roosens, Pieter-Jan Ceyssens, Wesley Mattheus, Kevin Vanneste

**Affiliations:** 1 Transversal activities in Applied Genomics, Sciensano 54513, Brussels, Belgium; 2 Bacterial Diseases, Sciensano 54513, Brussels, Belgium; Maine Medical Center Department of Medicine, Portland, Maine, USA

**Keywords:** Nanopore sequencing, core genome multi-locus sequence typing (cgMLST), public health, whole-genome sequencing, outbreak investigation, cluster detection

## Abstract

Core-genome multi-locus sequence typing (cgMLST) is a well-established and standardized method for genomics-based cluster detection and phylogenomic analysis of bacteria. The reduced error rate of Oxford Nanopore Technologies (ONT) R10 sequencing has prompted many laboratories to explore incorporating this technology into their activities. However, conflicting reports exist on the performance of ONT R10 sequencing for cgMLST analysis. This study evaluates the suitability of ONT R10 data for cgMLST allele calling and cluster detection for bacterial outbreak investigation. ONT and Illumina sequencing data were generated for 24 *Neisseria meningitidis* and 24 *Salmonella enterica* isolates. For ONT, both the rapid barcoding kit (RBK) and the rapid PCR barcoding kit (RPB) were used. The percentage of loci called in the ONT-only assemblies was very high for both species. However, the proportion of mismatched alleles to the hybrid assemblies was substantially higher for the *Neisseria* ONT-only assemblies with the RBK kit, resulting in incorrect cluster assignments. The large majority of these mismatched alleles were due to incorrect base calls at methylated positions, which did not affect the ONT data generated using the RPB kit or any of the *Salmonella* ONT-only assemblies. In conclusion, ONT R10 sequencing shows great potential as a viable method for cgMLST analysis, but methylation-related errors can affect performance for certain species and strains. When properly corrected for, ONT R10 had the same performance for cgMLST analysis as Illumina, and both could be used interchangeably. These results support the integration of ONT R10 sequencing into routine public health and clinical workflows.

**IMPORTANCE:**

This study evaluates the suitability of Oxford Nanopore Technologies R10 sequencing for core-genome multi-locus sequence typing (cgMLST), a widely used method in (clinical) outbreak investigation and bacterial strain tracking. We have sequenced 24 *Neisseria meningitidis* and 24 *Salmonella enterica* strains, including confirmed outbreak cases, using Illumina and ONT R10 sequencing to evaluate the performance for cgMLST analysis. We used a PCR-based and native barcoding protocol for the ONT sequencing, which enabled us to demonstrate a substantial species-dependent impact of methylation-related errors on the performance. However, we demonstrate that when these errors are properly addressed, ONT R10 can be used for accurate cgMLST-based clustering, including integration with strains sequenced using Illumina. Our findings support the use of ONT R10 as an alternative to Illumina sequencing for cgMLST analysis in routine public health practice.

## INTRODUCTION

Genomic surveillance and outbreak investigation of microbial pathogens is an essential task for many public health and clinical laboratories. Historically, multi-locus sequence typing (MLST) has been widely used as a typing method for bacteria. In MLST, a small set of housekeeping genes is sequenced with Sanger sequencing to type bacterial isolates based on their allelic profiles, by comparing against a harmonized reference database where each different sequence receives a different identifier, providing a standardized way to study genetic diversity and epidemiology (1). Allele sequences and MLST profiles are maintained in public repositories such as PubMLST, which hosts databases for over 130 species and genera (2). Historically, the lower throughput of Sanger sequencing has limited MLST to a few loci. The onset of whole-genome sequencing (WGS) has rendered it possible to scale MLST to hundreds or thousands of loci across the genome, greatly increasing the resolution. Consequently, core-genome MLST (cgMLST) and whole-genome MLST (wgMLST) schemes were developed for many species (3, 4). In cgMLST, the scheme is restricted to core loci shared by the majority of strains within a species or genus (typically found in ≥95% of strains). By contrast, wgMLST includes all annotated loci in the genome, including both core and accessory genes (i.e., genes that are not universally present in all members of a species). cgMLST or wgMLST allele calls can be used to build phylogenetic trees by generating matrices of allelic differences, which can then be analyzed using algorithms such as Minimum Spanning Tree (MST) construction to infer phylogenomic relationships (5). Due to its high resolution and ease of standardization, cgMLST has become a standard method for bacterial outbreak investigation with several case studies demonstrating its added value (6–8).

Recently, long-read Oxford Nanopore Technologies (ONT) sequencing has gained prominence as an alternative to short-read Illumina sequencing for WGS-based characterization of microbes (9, 10). ONT sequencing measures the electric current flowing through nanopores as DNA or RNA molecules pass through them. These raw electrical signals can then be translated into sequences using machine-learning-based methods. This process, known as basecalling, relies on specially trained models, which are made available by ONT and regularly updated. ONT sequencing has several characteristics that could render it particularly suitable for analyses such as outbreak investigation. Turnaround time can be minimized by live basecalling, speeding up data availability. In addition, the longer reads can resolve complex repeat regions, resulting in (nearly) complete assemblies, providing valuable insight into genomic organization (11). Less fragmented assemblies could also potentially result in more typed loci, as there is less chance of assembly fragmentation within loci (12). However, the relatively high error rate compared to short-read sequencing has made it challenging to apply it to cgMLST, as a single error in a cgMLST locus will result in a different allele call (13). In addition, ONT sequencing can require substantial data storage and computing resources, especially when using the most accurate basecalling models.

The error rate of ONT sequencing has been reduced substantially with the release of the R10 chemistry in 2022, also referred to as “Q20 sequencing” (14). Recent studies have shown that ONT R10 sequencing enables the reconstruction of near-perfect genome assemblies for various bacterial species, without the need for additional short-read sequencing (13–15). In theory, this makes ONT R10 sequencing well-suited for cgMLST analysis. However, several recent studies have reported errors related to methylation, which affects the electrical signal and basecalling accuracy, potentially leading to inaccuracies in the resulting consensus sequence (16–20) and, consequently, impacting cgMLST allele calling. One potential solution is to use the rapid PCR barcoding (RPB) kit to remove epigenetic modifications prior to sequencing, thereby preventing methylation-related errors (16). However, this approach is more labor-intensive compared to the rapid barcoding kit (RBK) and inherently limits the resulting read lengths to the size of the amplification range. Alternative strategies have been proposed to mask the methylated positions, but these approaches are unsuitable for allele calling because calling cgMLST loci requires complete allele sequences (16, 19). In May 2024, ONT released updated basecalling models (v5), specifically designed to improve the accuracy of base calls at methylated positions in bacteria. In addition, the “bacteria” preset has recently been added to the medaka long-read polishing software (available at https://github.com/nanoporetech/medaka), which also aims to correct errors caused by methylation in bacterial genomes. However, the impact of methylation is still unclear, as some studies comparing Illumina and ONT sequencing have found no discernible differences (13, 21), while others have reported problems with methylated positions affecting basecalling accuracy and subsequent (cg)MLST allele calling in various species (16, 17, 19, 22). In addition, ONT has been reported to suffer from lower accuracy in homopolymer regions compared to Illumina, resulting in indels which could further affect downstream analysis (23). The effects of these errors on the subsequent outbreak investigation based on observed clusters in the phylogenomic analysis remain poorly understood.

In this study, we evaluated the feasibility of using ONT R10 sequencing as an alternative to Illumina sequencing for cgMLST-based phylogenomic investigation and cluster detection for *Neisseria meningitidis* and *Salmonella enterica*, two species that have been extensively studied using cgMLST (4, 24). Hybrid assemblies were used as reference data to assess the performance of allele calling and clustering using ONT data base-called with the latest super high accuracy (SUP) model (v5). The potential effect of methylation on the accuracy of cgMLST was assessed by (i) sequencing strains using both the RBK and RPB kits and (ii) performing methylation calling at the discrepant sites within the cgMLST loci. In addition, the effect of long-read polishing was evaluated. This study demonstrates that when methylation effects are properly accounted for, ONT R10 can be used interchangeably with Illumina sequencing for accurate cgMLST calling and cluster detection.

## MATERIALS AND METHODS

### Data set

#### Selection of bacterial isolates and DNA extraction

The performance evaluation was performed on 24 *Neisseria meningitidis* and 24 *Salmonella enterica* isolates, of which an overview is provided in Table 1. The majority of *Neisseria* samples (*n* = 18) were collected between 2016 and 2023 by the Belgian National Reference Center (NRC) for *Neisseria meningitidis* (Sciensano, Brussels) in the context of their routine surveillance activities. This collection was supplemented with six isolates from the global collection maintained by the University of Oxford (24), originally sequenced for the validation of a bioinformatics workflow for the characterization of *Neisseria meningitidis* (25). These 24 isolates covered 12 different sequence types (STs), including ST11 (*n* = 5) and ST269 (*n* = 5), which are among the most common STs in Belgium (26). Two of the ST269 isolates were collected from a confirmed outbreak cluster. The data set also contained three additional ST269 isolates from lineages circulating in the same region around the time of the outbreak, but not part of the outbreak. In addition, clinical duplicates from two patients were included.

**TABLE 1:** Overview of the isolates in the study^*a*^

Genus	Isolate	Serogroup/serovar	ST	cgMLST cluster	Additional information
*Neisseria*	S18BD02235	MenB	ST269	2	Local outbreak—part of cluster
*Neisseria*	S19BD00002	MenB	ST269	2	Local outbreak—part of cluster
*Neisseria*	S18BD01608	MenB	ST269		Local outbreak—unrelated regional circulating lineage
*Neisseria*	S18BD08604^*b*^	MenB	ST269		Local outbreak—unrelated regional circulating lineage
*Neisseria*	S16BD06814	MenB	ST269		Local outbreak—unrelated regional circulating lineage
*Neisseria*	Z1269	MenA	ST4		Global Reference Collection
*Neisseria*	Z1534	MenA	ST21		Global Reference Collection
*Neisseria*	Z4678	MenB	ST19		Global Reference Collection
*Neisseria*	Z5037	MenC	ST2		Global Reference Collection
*Neisseria*	Z6431	MenB	ST28		Global Reference Collection
*Neisseria*	Z6422	MenW	ST45		Global Reference Collection
*Neisseria*	S18BD08034	MenA	ST7		EQA^*c*^ reference strain
*Neisseria*	S19BD00230	MenB	ST457	4	Clinical duplicate—Patient A
*Neisseria*	S19BD00371	MenB	ST457	4	Clinical duplicate—Patient A
*Neisseria*	S18BD00375	MenY	ST11846	1	Clinical duplicate—Patient B
*Neisseria*	S18BD00609	MenY	ST11846	1	Clinical duplicate—Patient B
*Neisseria*	S23BD02713	MenW	ST9316		Routine surveillance—MenW Walloon lineage
*Neisseria*	S23BD06477	MenW	ST9316		Routine surveillance—MenW Walloon lineage
*Neisseria*	S22BD09725	MenW	ST9316		Routine surveillance—MenW Walloon lineage
*Neisseria*	S20BD05309	MenW	ST11		Routine surveillance—MenW UK lineage
*Neisseria*	S18BD02673	MenW	ST11		Routine surveillance—MenW UK lineage
*Neisseria*	S23BD04233	MenW	ST11		Routine surveillance—MenW UK lineage
*Neisseria*	S19BD08966	MenW	ST11	3	Routine surveillance—MenW Hajj lineage
*Neisseria*	S18BD07975	MenW	ST11	3	Routine surveillance—MenW Hajj lineage
*Salmonella*	S23BD05331	Dublin	ST10	5	EQA reference strain—Part of cluster A
*Salmonella*	S23BD05333	Dublin	ST10	5	EQA reference strain—Part of cluster A
*Salmonella*	S23BD05337	Dublin	ST10	5	EQA reference strain—Part of cluster A
*Salmonella*	S23BD05340	Dublin	ST10	5	EQA reference strain—Part of cluster A
*Salmonella*	S23BD05338	Dublin	ST10		EQA reference strain
*Salmonella*	S23BD08066	Monophasic Typhimurium	ST34	6	Clinical duplicate—Patient A
*Salmonella*	S23BD09059	Monophasic Typhimurium	ST34	6	Clinical duplicate—Patient A
*Salmonella*	S22BD04539	Monophasic Typhimurium	ST34		EQA reference strain
*Salmonella*	S22BD04543	Senftenberg	ST14		EQA reference strain
*Salmonella*	S22BD05218	Enteritidis	ST11	3	EQA reference strain—Part of cluster B
*Salmonella*	S22BD05221	Enteritidis	ST11	3	EQA reference strain—Part of cluster B
*Salmonella*	S22BD05226	Enteritidis	ST11	3	EQA reference strain—Part of cluster B
*Salmonella*	S22BD05220	Enteritidis	ST11		EQA reference strain
*Salmonella*	S22BD01093	Monophasic Typhimurium	ST34	1	International outbreak cluster C—sub-cluster 1
*Salmonella*	S22BD01190	Monophasic Typhimurium	ST34	1	International outbreak cluster C—sub-cluster 1
*Salmonella*	S23BD06998^*b*^	Monophasic Typhimurium	ST34		International outbreak C—Outlier
*Salmonella*	S22BD01330	Monophasic Typhimurium	ST34	2	International outbreak cluster C—sub-cluster 2
*Salmonella*	S22BD01427	Monophasic Typhimurium	ST34	2	International outbreak cluster C—sub-cluster 2
*Salmonella*	S24BD02659	Monophasic Typhimurium	ST34		International outbreak C—Outlier
*Salmonella*	S23BD05085	Enteritidis	ST11	4	International outbreak D—Cluster
*Salmonella*	S22BD04657	Enteritidis	ST11		International outbreak D—Outlier
*Salmonella*	S24BD01743	Enteritidis	ST11	4	International outbreak D—Cluster
*Salmonella*	S24BD01974	Enteritidis	ST11		International outbreak D—Outlier
*Salmonella*	S22BD04540	Heidelberg	ST15		EQA reference strain

^*a*^ Overview of the strains used in this study. The first and second columns contain the genus and isolate name, respectively. The third column contains the serogroup for the *Neisseria* strains and the serovar for the *Salmonella* strains. The fourth column contains the sequence type. The fifth column contains the cluster assignment based on cgMLST analysis using the strict clustering threshold (see “Phylogenetic tree construction and clustering”). Note that the *Salmonella* S23BD05085 and S24BD01743 isolates clustered according to cgMLST, while the metadata stated they were unrelated. The allelic distance between the two isolates was 5, which is the cut-off for the strict clustering. In practice, this would prompt an investigation using epidemiological data to verify the potential relationship between the two strains. The last column contains additional metadata for the isolates, including membership of known clusters.

^*b*^ The ONT data for the *Neisseria* S18BD08604 and *Salmonella* S23BD06998 isolates generated using the RBK kit were low quality, and these data sets were not retained for the subsequent performance evaluation.

^*c*^ EQA, external quality assessment.

For *Salmonella,* the isolates were collected by the Belgian NRC for *Salmonella* and *Shigella* spp. between 2022 and 2024, complemented by several isolates from external quality assessments (EQAs). This data set included five STs, the most common being monophasic *Salmonella* ser. Typhimurium ST34 (*n* = 9) and *Salmonella* ser. Enteritidis ST11 (*n* = 8). The data set contained isolates from various outbreaks: (i) a cluster of three related ST11 isolates from an EQA; (ii) two sub-clusters of an international foodborne outbreak, as well as outliers for both sub-groups; (iii) an isolate from another international foodborne outbreak, as well as three outliers; and (iv) two clinical duplicates collected from routine surveillance.

For both bacterial species, isolates were cultured overnight in brain heart infusion broth at 37°C, and 1 mL was used for semi-automated DNA extraction using the MagCore Genomic DNA Bacterial kit (#502, Atrida, Amersfoort, The Netherlands) with a 100 µL elution volume, according to the manufacturer’s instructions.

#### Whole-genome sequencing

The ONT long-read libraries were prepared using the RBK v14 kit (SQK-RBK114-96) for sequencing native DNA and the ONT RPB v14 kit (SQK-RPB114-24) for sequencing amplified DNA, that is, DNA without epigenetic markers, according to the manufacturer’s instructions. Libraries were loaded onto R10.4.1 flow cells (FLO-MIN114) and sequenced for 72 h on a GridION instrument. A single flow cell was used per barcoding kit and species, totaling four flow cells for the ONT data. The Illumina sequencing is documented in the Supplementary Material.

### Data preprocessing, filtering, and *de novo* assembly

Basecalling for the ONT data was performed with Dorado v0.7.0 using the super accurate (SUP) model v5.0.0 (dna_r10.4.1_e8.2_400bps_sup@v5.0.0) and the “--no-trim” option enabled. The base called reads were then de-multiplexed using the “dorado demux” command specifying the barcoding kit with the “--kit-name” parameter. Reads were extracted from the BAM files using the “fastq” command of samtools v1.17 (27) and filtered using Seqkit v2.8.2 to remove reads shorter than 1 kb or with a median quality of less than 10 (28). The filtered reads were assembled *de novo* using Flye v2.9.4 (29) providing the input with the “--nano-corr” option and the “--genome-size” parameter set to 2,200,000 and 5,000,000 for the *Neisseria* and *Salmonella* data sets, respectively. The high-coverage *Neisseria* S16BD06814 RBK and S18BD02673 RBK data sets failed to assemble with these options and were restarted with the “--asm-coverage” parameter set to 50. QUAST v5.2.0 (30) was used to assess the quality of the assemblies, with both the assemblies and the reads provided as input, the latter using the “--nanopore” option. Additional assemblies were generated by long-read polishing of the Flye assemblies with the filtered reads using medaka v1.12.0 (available at https://github.com/nanoporetech/medaka). The “medaka_consensus” script was used with the model parameter set to “r1041_e82_400bps_sup_v5.0.0” and other options left at their default values. In this manuscript, the term “polished ONT assemblies” refers to ONT-only assemblies that were polished using long reads with Medaka. While the term “polished assemblies” is sometimes also used in the literature to refer to hybrid assemblies polished with short and/or long reads, we will consistently use “hybrid assemblies” throughout. Information on the pre-processing of the Illumina data and hybrid assembly methods can be found in the Supplementary Material.

### cgMLST allele calling and clustering

#### Allele calling

Alleles were called as described previously (25), with blast (31) updated to v2.14.0, on the Illumina, unpolished and polished ONT, and hybrid assemblies. For the *Neisseria* data sets, the cgMLST v3 scheme from PubMLST (3), containing 1,329 loci, was used (accessed on September 11th, 2024). For *Salmonella*, the EnteroBase (4) cgMLST scheme, containing 3,002 loci, was used (accessed on September 11th, 2024). Alleles detected in the hybrid assembly data sets that aligned over the full length of an existing allele in the database with >99% nucleotide identity were considered valid novel alleles. Updated cgMLST schemes were constructed locally by introducing these novel alleles with temporary allele identifiers, an approach functionally equivalent to submitting the alleles to the underlying databases maintained by PubMLST and EnteroBase (32). These schemes were used for further processing.

#### Phylogenetic tree construction and clustering

Phylogenetic trees were constructed from the cgMLST allele calls using an in-house script written in Python (https://github.com/BioinformaticsPlatformWIV-ISP/mlst_phylogeny). The script first constructs an allele matrix, considering only perfect hits (i.e., full length and 100% nucleotide identity). Then, loci detected in <75% of data sets were removed from the phylogenetic analysis. Note that these loci were still retained for the performance evaluation described below. Pairwise cgMLST distances were extracted from this filtered matrix. MSTs were then constructed using GrapeTree v2.2 (5) with the method parameter set to “MSTreeV2.”

Clusters were defined using strict and loose cluster definition thresholds based on pairwise allele distances. For *Neisseria*, the loose threshold, defined as possibly epidemiologically linked, and the strict threshold, representing core clusters, were set at seven and four alleles, respectively (33). For *Salmonella*, loose and strict thresholds of ten and five alleles, respectively, were used. These thresholds were determined based on international guidelines (33) and retrospective evaluation of internal data sets and associated metadata.

### Performance evaluation

#### Mismatches to hybrid assembly

The cgMLST allele calls for all loci for ONT assemblies were evaluated using the results of the corresponding hybrid assemblies for all individual isolates as ground truth. For instance, the allele calls in the unpolished and polished assemblies from ONT data generated with the RBK kit were compared with the allele calls in the hybrid assemblies generated using the same ONT data. The results of the Illumina data sets were compared to the results of both hybrid assemblies (i.e., generated with the RBK and RPB ONT data). Alleles were considered a match if the same allele was detected as a perfect hit in both data sets, that is, defined here as a full-length hit with perfect nucleotide identity. If no perfect hit was found in either data set, the locus was also considered a match (i.e., a locus that is absent in both replicates). All other cases were classified as mismatches. Note that this strategy differs from the “MSTreeV2” tree-building algorithm, where a missing allele in one of the data sets is not considered a mismatch but rather is treated as missing data, and therefore does not contribute to the distance between isolates in the MST.

#### Impact on clustering

**Fig 1:**
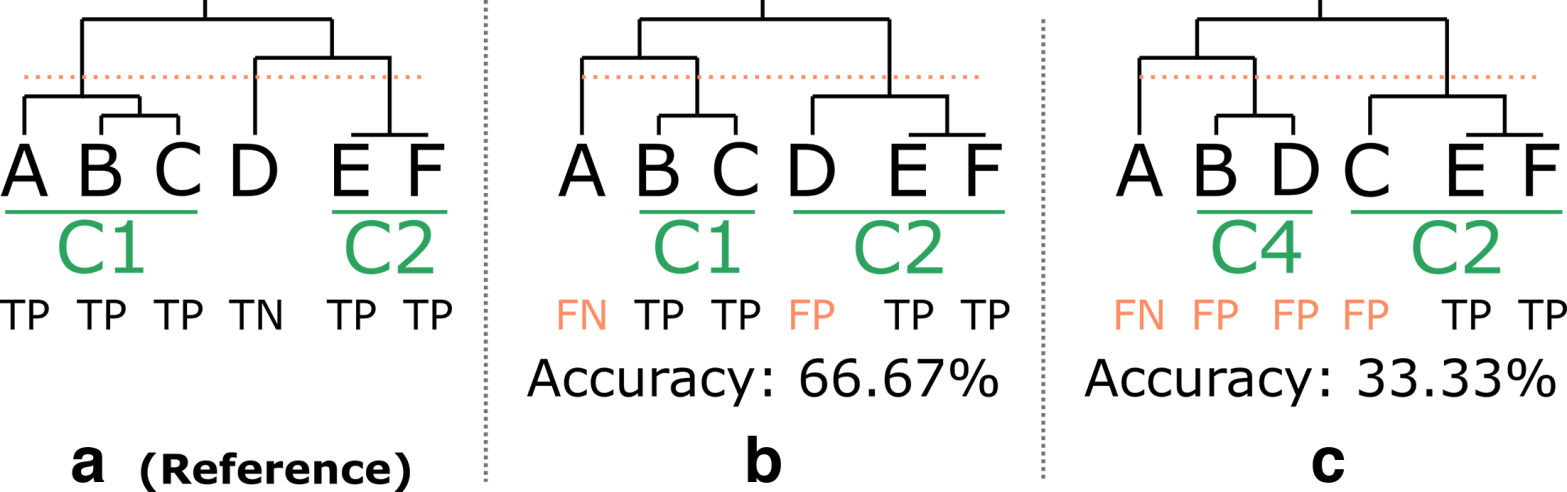
Overview of clustering definitions. This figure shows an example phylogeny to illustrate the definitions used to evaluate clustering performance. Figure A shows the reference phylogeny (based on the hybrid assemblies). Figures B and C show hypothetical phylogenies and the corresponding classification based on using ONT data. Isolates with a distance below the clustering threshold (indicated by an orange horizontal line) are grouped into clusters. Cluster labels are assigned based on the reference phylogeny: if more than half of the isolates within a cluster share the same label, the cluster is assigned that corresponding label. The detected clusters are shown in green. Accuracy was calculated by dividing the number of TP and TN by the total number of isolates and is given at the bottom of the figure. The left example (**a**) shows the reference topology and cluster assignments with one cluster of three isolates (i.e., A, B, and C) and a second cluster of two isolates (i.e., E and F). Isolates in a cluster are classified as TP, and isolates outside the clusters are classified as TN. In the second example (**b**), isolate A is classified as an FN because it is not assigned to cluster 1. Isolates B and C are classified as TP because they are correctly assigned to cluster 1. Isolate D is classified as an FP because it is incorrectly clustered with isolates E and F in cluster 2. Isolates E and F are correctly assigned to cluster 2 and classified as TP. In the right example (**c**), isolate A is still classified as an FN. Isolates B and D cluster together, but this cluster does not match the reference cluster for either isolate and is therefore classified as FP. Isolate C is incorrectly assigned to cluster 2 and is therefore classified as an FP. Isolates E and F are in the correct cluster and are classified as TP. Abbreviations: true positive (TP), true negative (TN), false negative (FN), false positive (FP), reference (ref.).

The effect of sequencing technology and protocol on the clustering was assessed by constructing phylogenies using the strategy described in “Phylogenetic tree construction and clustering,” above. Both ONT barcoding protocols were evaluated with and without polishing. The assemblies generated using the Illumina data were analyzed using the same methodology. Analogous to the allele calling evaluation (see “Mismatches to hybrid assembly,” above), results of cluster detection in the hybrid data sets, using the thresholds described in “Phylogenetic tree construction and clustering,” above, were considered the “ground truth” for comparing either Illumina and ONT data sets. The evaluation was performed separately for the loose and strict thresholds. Each isolate was classified according to the following definitions: true positives (TP) were defined as isolates that clustered in the reference phylogeny and were assigned to the correct cluster in the tested phylogeny; true negatives (TN) were defined as isolates that did not cluster in the reference phylogeny and were not assigned to any cluster in the tested phylogeny; (FP) were defined as isolates that did not cluster in the reference phylogeny and were incorrectly assigned to a cluster or were assigned to the wrong cluster in the tested phylogeny; (FN) were defined as clustered isolates in the reference phylogeny that were not assigned to the correct cluster in the tested phylogeny. Several examples are provided in Fig. 1. Accuracy was then calculated by dividing the number of TPs and TNs by the total number of isolates in the phylogeny.

#### Tree and distance matrix similarity metrics

The similarity of the resulting phylogenetic trees and allele-based distance matrices was evaluated using several metrics that compared them to the results of their respective hybrid assemblies (see “Data preprocessing, filtering, and *de novo* assembly,” above). The following metrics were evaluated: (i) Robinson-Foulds distance, which quantifies differences by counting mismatches splits (34), (ii) Kendall-Colijn distance, which accounts for both the topology and branch lengths (35), and (iii) the Pearson pairwise correlation of the allele distance matrices, which measures the similarity of all pairwise distances between two matrices. Unweighted Robinson-Foulds distances were calculated using the DendroPy Python package v5.0.1 (36) in Python v3.10. Kendall-Colijn distances were calculated using the R script provided by Katz et al. (37) in R v4.3.1 with the lambda parameter set to 0.5. The Pearson pairwise correlation was calculated using the “pearsonr” function of the SciPy Python package v1.14.0 (38) in Python v3.10.

#### Combining ONT R10 with Illumina sequencing

Given that many laboratories have relied on short-read sequencing to build extensive collections of WGS data sets and cgMLST profiles, we also evaluated the integration of ONT R10 and Illumina sequencing specifically for phylogenetic tree construction and cluster detection (9). First, we used the methodology detailed in “Mismatches to hybrid assembly,” above, with the Illumina assemblies as reference standard to determine the number of allele mismatches with the ONT data sets. These allele distances between the Illumina and ONT data sets for the same samples were used to assess whether the data generation method could lead to missed clusters. Second, we constructed mixed phylogenies combining the Illumina and ONT R10 data sets into the same phylogeny to assess how potential inconsistencies between the two technologies might affect the resulting phylogenies.

### Investigation of mismatches and methylation calling

The mismatches in the discrepant cgMLST loci were investigated by identifying SNPs and indels between the hybrid and unpolished assemblies using dnadiff from the MUMmer4 v4.0.0rc1 package (39) with default options. The SNPs and indels within the cgMLST loci were extracted using a custom script. In addition, basecalling was repeated as described in “Data preprocessing, filtering and *de novo* assembly,” above, but with the “4mC_5mC” and “6mA” models added to perform methylation calling. The resulting reads were filtered as described previously and mapped to the corresponding hybrid assemblies using Minimap2 v2.26 (40) with the “-ax map-ont” and “-y” (i.e., to preserve methylation tags) options enabled. Modkit v0.3.1 (https://github.com/nanoporetech/modkit) was then used to call methylation. Assuming that sequencing errors are more likely to occur at methylated sites, methylation calling may be hindered by incorrectly called bases. For example, at a methylated cytosine site in the genome, 4mC or 5mC methylation can only be called on bases that are correctly identified as cytosine, which may be a minority at that position. To account for this, we applied relatively lenient filtering criteria: at least two reads with the corresponding base call and at least 2 out of 3 (i.e., 66.67%) of those classified as methylated. The SNPs detected by dnadiff and the methylation calls were then cross-checked. To account for potential basecalling inaccuracies due to nearby methylation, the positions immediately flanking the mutation were also checked. For the indels that were identified by dnadiff, a custom script was used to assess whether they were located within homopolymers. Erroneous indels were classified as homopolymer-related errors if they occurred in stretches of six or more consecutive identical bases.

## RESULTS

### WGS data quality and yield

Read statistics from four ONT runs (RBK and RPB protocols for *Neisseria* and *Salmonella*) showed similar total yields. However, the median read lengths before filtering were shorter with the RBK kit due to many short reads. After filtering, read lengths were similar for both kits for the *Neisseria* data sets, but remained higher for the RBK kit in the *Salmonella* data sets. For both species, RBK data sets showed higher N50 values and less fragmented assemblies, with more complete circular chromosomes, which is likely due to the longer reads. By contrast, the RPB protocol produced slightly higher read quality and more consistent coverage across isolates. All data sets were retained for the subsequent analyses, except for the *Neisseria* S18BD08604 RBK and *Salmonella* S23BD06998 RBK, for which the data quality was deemed insufficient. All Illumina data sets passed the quality checks and were retained for subsequent analysis. Detailed results are provided in the Supplementary Material.

### cgMLST allele calling and clustering

#### cgMLST allele calling

As some of these genomes were not previously deposited in public databases, the analysis started by identifying high-quality alleles which were not yet present in the underlying cgMLST databases. For *Neisseria,* a total of 157 novel cgMLST alleles were detected across the hybrid assemblies for all isolates. Nearly all of these novel alleles were consistent across the hybrid assemblies for both protocols (i.e., they were detected as perfect hits in both hybrid assemblies). For *Salmonella*, 10 novel alleles were detected across the hybrid assemblies for all isolates. All novel detected alleles were then introduced into local copies of the corresponding cgMLST schemes for both species, and the cgMLST analyses were re-run using these updated schemes.

The percentage of cgMLST loci called afterwards for all data sets and assembly approaches is shown in Table 2. For *Neisseria*, the median percentage of loci called in the hybrid assembly data sets was similar for the RBK and RPB kits with 99.85% and 99.81%, respectively. Interestingly, the median percentage of loci detected in the Illumina data sets was slightly lower at 99.77%. Long-read polishing with Medaka had no impact on the percentage of perfect hits in the RPB-only assemblies. However, for the RBK-only assemblies, polishing slightly reduced the median percentage of loci detected. For *Salmonella*, the percentage of loci called as perfect hits in the hybrid assemblies was similar, with 97.90% and 97.88% for the RBK and RPB kits, respectively. The median percentage of loci detected in the Illumina data sets was comparable at 97.88%. The results for the unpolished and polished ONT-only assemblies were also comparable, ranging from 97.83% to 97.87%. In general, the fraction of alleles called perfect hits was substantially lower for *Salmonella* compared to *Neisseria*. Figure S6 provides a breakdown of the uncalled cgMLST loci in the hybrid assemblies. For *Salmonella*, the main cause of uncalled alleles was multi-hits, that is, multiple alleles with the same alignment statistics where the workflow could not select a best match. A schematic representation of a multi-hit is provided in Fig. S7. These multi-hits were restricted to 112 loci, corresponding to <4% of the loci in the scheme. As multi-hits were consistently detected across sequencing and assembly methods and were very rare in the *Neisseria* data sets, we suspect this may be a consequence of problematic alleles in the *Salmonella* cgMLST scheme rather than the allele calling method or data quality.

**TABLE 2:** Percentage of cgMLST loci detected^*a*^

Genus	Sequencing technology and protocol	Assembly method	Median no. of cgMLST loci detected (%)
*Neisseria*	RBK + Illumina	Hybrid	1,327/1,329 (99.85)
*Neisseria*	RPB + Illumina	Hybrid	1,326.5/1,329 (99.81)
*Neisseria*	RBK	ONT-only + medaka polishing	1,322/1,329 (99.47)
*Neisseria*	RPB	ONT-only + medaka polishing	1,327/1,329 (99.85)
*Neisseria*	RBK	ONT-only unpolished	1,323.5/1,329 (99.59)
*Neisseria*	RPB	ONT-only unpolished	1,327/1,329 (99.85)
*Neisseria*	Illumina	Illumina-only	1,326/1,329 (99.77)
*Salmonella*	RBK + Illumina	Hybrid	2,939/3,002 (97.90)
*Salmonella*	RPB + Illumina	Hybrid	2,938.5/3,002 (97.88)
*Salmonella*	RBK	ONT-only + medaka polishing	2,938/3,002 (97.87)
*Salmonella*	RPB	ONT-only + medaka polishing	2,937.5/3,002 (97.85)
*Salmonella*	RBK	ONT-only unpolished	2,937/3,002 (97.83)
*Salmonella*	RPB	ONT-only unpolished	2,938/3,002 (97.87)
*Salmonella*	Illumina	Illumina-only	2,938.5/3,002 (97.88)

^*a*^ This table lists the median percentage of cgMLST loci detected for each sequencing technology and protocol, and assembly method. The schemes for *Neisseria* and *Salmonella* contain 1,329 and 3,002 loci, respectively. Note that only perfect hits are considered (i.e., full length and perfect nucleotide identity). The columns list the genus name, sequencing technology & protocol, assembly method, and median percentage of cgMLST loci detected.

#### Clustering

**Fig 2:**
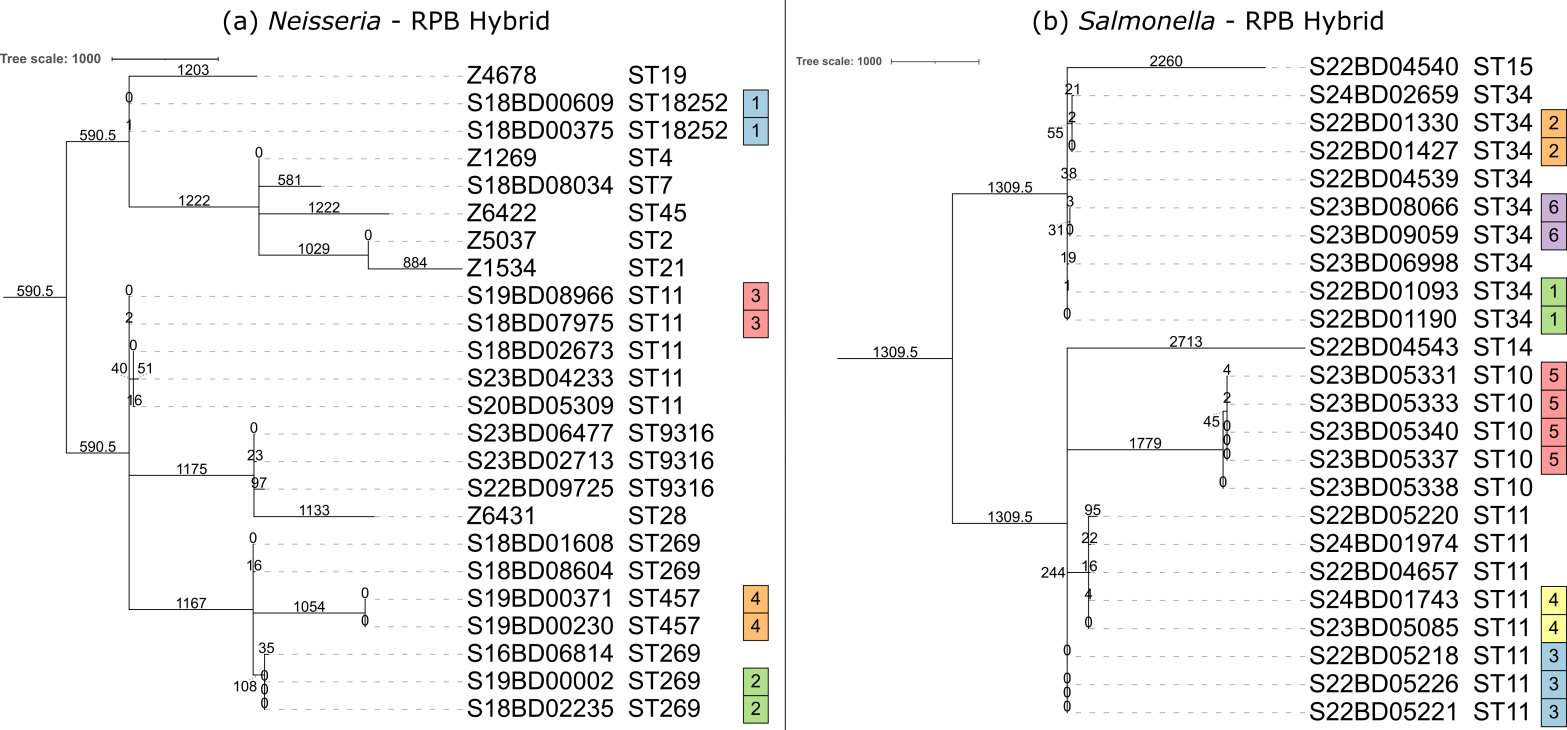
Reference phylogenies and clustering. These figures show the reference minimum spanning tree (MST) phylogenies, displayed as phylogenetic trees, for the (a) *Neisseria* and (b) *Salmonella* datasets, constructed from the RPB hybrid assemblies. The annotations are from left to right: isolate name, sequence type (ST), and cluster membership. Clusters were defined as isolates that clustered within four and five alleles of each other for *Neisseria* and *Salmonella*, respectively (i.e., the strict thresholds). The loose thresholds are not shown as the obtained clusters were identical. The results for the hybrid assemblies generated with the RBK data sets were very similar and are shown in Fig. S8. The scale bar is expressed as number of cgMLST differences, and branch lengths are indicated on branches. The phylogenetic tree was midpoint-rooted, resulting in some branch lengths being represented as decimals. The visualizations were created using iToL (41).

To assess the clustering performance of the ONT and Illumina data, we first established the ground truth by performing clustering on the cgMLST results on the hybrid assemblies. The cgMLST-based phylogenies for both species and kits, constructed from the hybrid assemblies, are shown in Fig. 2 for the RPB kit and in Fig. S8 for the RBK kit. The phylogenies were very similar, regardless of the barcoding kit used. The clusters detected using the loose and strict thresholds were identical for both species and were mostly consistent with the available metadata (Table 1). For *Neisseria*, four clusters were detected, each consisting of two isolates. Two clusters corresponded to ST457 and ST11846, which were the only isolates of their respective STs. These two clusters correspond to clinical duplicates collected from two different patients. The other two clusters were part of larger groups: ST11 and ST269, both with a total of five isolates. The ST11 cluster consisted of two strains of the serogroup W Hajj lineage collected during routine surveillance and not found to be part of an outbreak cluster. The two ST269 isolates in the corresponding cluster were confirmed as part of an outbreak and clustered separately from the other ST269 isolates circulating in the region during the outbreak. The pairwise distances for the ST11 and ST11846 clusters were two alleles, and the isolates in the ST269 and ST457 clusters had identical cgMLST profiles to each other (Fig. S9). For *Salmonella*, four clusters of two isolates were found (three assigned to ST34 and one to ST11), a cluster of three ST11 isolates, and a cluster of four ST10 isolates (Fig. S10). The ST10 cluster of four isolates (i.e., cluster 5) and the ST11 cluster of three isolates (i.e., cluster 3) both consist of EQA samples and were in agreement with the provided metadata, meaning that the isolates with known cluster association clustered together, apart from the unrelated isolate(s). Three of the two-isolate clusters were assigned to ST34, which had a total of nine isolates. One of the clusters corresponded to clinical duplicates from the same patient (i.e., cluster 6) and the other two to confirmed sub-clusters within an international foodborne outbreak (i.e., clusters 1 and 2). The last cluster consisted of two ST11 isolates, including a confirmed outbreak strain and an outlier from the same outbreak. This cluster assignment was not consistent with the metadata, but the pairwise allele distance was five, which is the value of the strict threshold for cluster definition. Overall, the pairwise distances between *Salmonella* isolates in the same cluster ranged from zero to five alleles. The same clusters were obtained using the loose and strict cluster definition thresholds.

### Performance evaluation

#### cgMLST mismatches of the ONT and Illumina data sets

**Fig 3:**
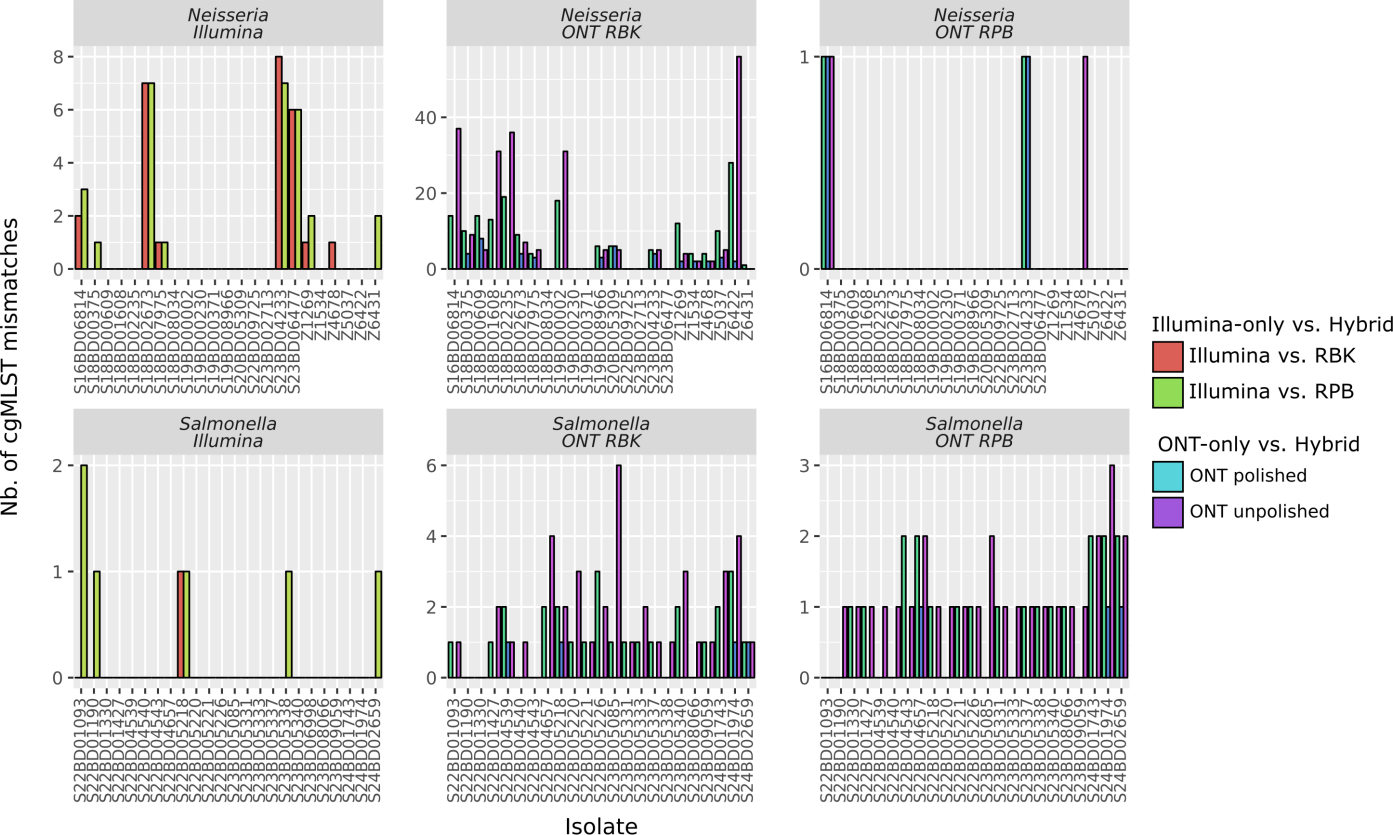
Mismatched cgMLST loci in the Illumina- and ONT-only assemblies compared to the hybrid assembly. Number of cgMLST locus mismatches between the Illumina- and ONT-only assemblies and their corresponding reference hybrid assemblies. The x-axis represents individual isolates, while the y-axis shows the number of cgMLST mismatches between the assemblies and the corresponding hybrid assemblies. Note that the scale of the y-axis varies between sub-plots to accommodate the different value ranges. The comparison of the Illumina assemblies with the RBK and RPB hybrid assemblies is shown in red and green, respectively. The results for the unpolished and medaka-polished ONT-only assemblies are shown in purple and blue, respectively. The median, minimum, and maximum values per kit are listed in Table S6. The ONT data for the *Neisseria* S18BD08604 and *Salmonella* S23BD06998 isolates generated using the RBK kit were low quality and these isolates were not included.

A visualization of the number of mismatches for each ONT- and Illumina-only data set compared to the corresponding hybrid assemblies is provided in Fig. 3. For *Neisseria*, the median number of mismatches was five and six for the unpolished and polished RBK kit assemblies, respectively. The variability between isolates was considerable, with five isolates having more than 30 mismatches in the unpolished ONT-only assemblies and six isolates having none. The number of mismatches with the RPB kit was much lower, with a median of zero and a maximum of one allele for both the unpolished and polished ONT-only assemblies. For *Salmonella*, the median number of mismatches was one for both kits with and without polishing. The total number of mismatches on the unpolished ONT-only assemblies was slightly higher for the RBK kit (*n* = 41) compared to the RPB kit (*n* = 29), but overall the number of mismatches was comparable. The maximum number of mismatches was six for the RBK kit and three for the RPB kit, and in contrast to the *Neisseria* results with the RBK kit, there were no extreme outliers. Mismatches occurred much more frequently in certain loci, as illustrated in Fig. S11. For example, in the *Salmonella* STMMW_17581 locus, which did not correspond to the hybrid assemblies in 18 of the 23 unpolished RBK assemblies and in 23 of the 24 unpolished RPB assemblies, whereas the vast majority of loci showed no mismatches at all. More information about potential causes for mismatches in the ONT-only assemblies is available in section “Investigation of mismatches and methylation calling.”

For the Illumina data sets, the proportion of mismatches compared to the hybrid assemblies was comparable for both species, with a median of zero mismatches for *Neisseria* and *Salmonella* compared to both hybrid assemblies.

#### Cluster detection of the ONT and Illumina data sets

The performance expressed as accuracy of the cluster detection for each of the methodologies using the loose and strict clustering thresholds is shown in Table 3. The number of loci that passed the allele matrix filtering for each phylogeny is listed in Table S2. For both species and thresholds, the isolates sequenced by Illumina were always correctly classified into the reference cluster or as unrelated isolates. For the *Neisseria* isolates sequenced using ONT and the RPB kit, the correct clustering was obtained with and without polishing for both thresholds. The accuracy for the RBK kit and the strict threshold was 75%, regardless of polishing. Only the cluster containing S19BD00230 and S19BD00371 (i.e., cluster 4) was correctly identified in the RBK data set, while the six isolates of the other clusters did not group as expected (Fig. S9). No FP or FN clusters were detected, resulting in 18 out of 24 isolates (75%) being correctly assigned. Using the loose threshold, the cluster containing S18BD07975 and S19BD08966 (i.e., cluster 3) was also correctly identified, resulting in an accuracy of 83.33% (i.e., 20 out of 24 isolates correctly classified). For *Salmonella*, 100% accuracy was obtained for all methods with the loose threshold. For the strict threshold, 100% accuracy was obtained with the unpolished RPB kit assemblies and the polished RBK assemblies. For the polished RPB assemblies, the S23BD05085 and S24BD01743 isolates (i.e., cluster 4) did not cluster together as expected, due to a difference of six alleles (Fig. S10). In addition, the S23BD05331 isolate was not assigned to cluster 5, resulting in an accuracy of 87.5% (i.e., 21 out of 24 correct classifications). For the unpolished RBK assemblies, the S23BD05085 and S24BD01743 isolates (i.e., cluster 4) did not cluster together in the ONT phylogeny, resulting in an accuracy of 91.3% (i.e., 21 out of 23 correct classifications). The pairwise distance between these isolates was seven alleles in the ONT data sets, compared to four alleles in the hybrid assemblies.

**TABLE 3:** Clustering performance^*a*^

Genus	Method	Barcoding kit	Accuracy (%)	
			Strict threshold	Loose threshold
*Neisseria*	Hybrid	RPB	100.00	100.00
*Neisseria*	ONT only unpolished	RPB	100.00	100.00
*Neisseria*	ONT only + medaka polishing	RPB	100.00	100.00
*Neisseria*	Hybrid	RBK	100.00	100.00
*Neisseria*	ONT only unpolished	RBK	75.00	83.33
*Neisseria*	ONT only + medaka polishing	RBK	75.00	83.33
*Neisseria*	Illumina-only	-	100.00	100.00
*Salmonella*	Hybrid	RPB	100.00	100.00
*Salmonella*	ONT only unpolished	RPB	100.00	100.00
*Salmonella*	ONT only + medaka polishing	RPB	87.50	100.00
*Salmonella*	Hybrid	RBK	100.00	100.00
*Salmonella*	ONT only unpolished	RBK	91.30	100.00
*Salmonella*	ONT only + medaka polishing	RBK	100.00	100.00
*Salmonella*	Illumina-only	-	100.00	100.00

^*a*^ This table shows the accuracy of the clustering by comparing with the results obtained using the hybrid assemblies with the strict and loose cluster definition thresholds. Accuracy was calculated by dividing the number of TP and TN by the total number of observations, with the definitions for TP, TN, FP, and FN results provided in section “Impact on clustering.” A visual explanation of these definitions is provided in Fig. 1. The loose threshold was seven alleles for *Neisseria* and ten alleles for *Salmonella*. The strict threshold was four alleles for *Neisseria* and five alleles for *Salmonella*.

#### Tree and distance similarity metrics of the ONT and Illumina data sets

The Robinson-Foulds distance, Kendall-Colijn distance, and Pearson correlation metrics are listed in Table 4. For the *Neisseria* data sets, phylogenies generated from the RPB data sets closely matched the reference phylogeny, resulting in the lowest Robinson-Foulds and Kendall-Colijn distances and the highest Pearson correlation values. By contrast, Illumina-based phylogenies showed greater divergence from the reference phylogeny and its distance matrix. This divergence can be explained by a larger number of “true” mismatches where a different allele is called as a perfect hit, rather than a mismatch where one allele is missing in one of the two datasets, which was more common in the Illumina data sets compared to the RPB data sets. The phylogenies obtained with the RBK data sets differed the most from the reference, with much higher values of the Robinson-Foulds and Kendall-Colijn distances and much lower distance matrix correlation. For the *Salmonella* data sets, the phylogenies generated from the RPB data sets were also the most similar to the reference phylogeny. In contrast to *Neisseria*, the phylogenies obtained with the RBK data sets were more similar to the reference phylogeny than the results obtained with Illumina. The Pearson correlation coefficients between the distance matrices were very high, with values > 99.9% for all methods. For both species and kits, polishing slightly reduced the similarity to the reference but did not have a substantial impact.

**TABLE 4:** Tree-similarity metrics^*a*^

Genus	Method	Barcoding kit	Robinson-Foulds distance	Kendall-Colijn distance	Pearson correlation (%)
*Neisseria*	Hybrid	RPB	0	0	100
*Neisseria*	ONT-only unpolished	RPB	0	0	100
*Neisseria*	ONT-only + medaka polishing	RPB	1	1	100
*Neisseria*	Hybrid	RBK	0	0	100
*Neisseria*	ONT-only unpolished	RBK	9	4,095	99.03
*Neisseria*	ONT-only + medaka polishing	RBK	10	4,141	99.16
*Neisseria*	Illumina-only	-	2	37	100
*Salmonella*	Hybrid	RPB	0	0	100
*Salmonella*	ONT-only unpolished	RPB	1	15	100
*Salmonella*	ONT-only + medaka polishing	RPB	2	21	100
*Salmonella*	Hybrid	RBK	0	0	100
*Salmonella*	ONT-only unpolished	RBK	9	3770	100
*Salmonella*	ONT-only + medaka polishing	RBK	9	3770	100
*Salmonella*	Illumina-only	-	10	7,340	100

^*a*^ This table lists the similarity metrics for the phylogenies and distance matrices obtained with the different assemblies. The evaluated metrics are the unweighted Robinson-Foulds distance, the Kendall-Colijn distance with the lambda parameter set to 0.5, and the Pearson correlation of the allele distance matrices. The results on the hybrid assemblies were used as ground truth.

#### Combining ONT and Illumina data sets within the same phylogenetic analysis and cluster detection

**Fig 4:**
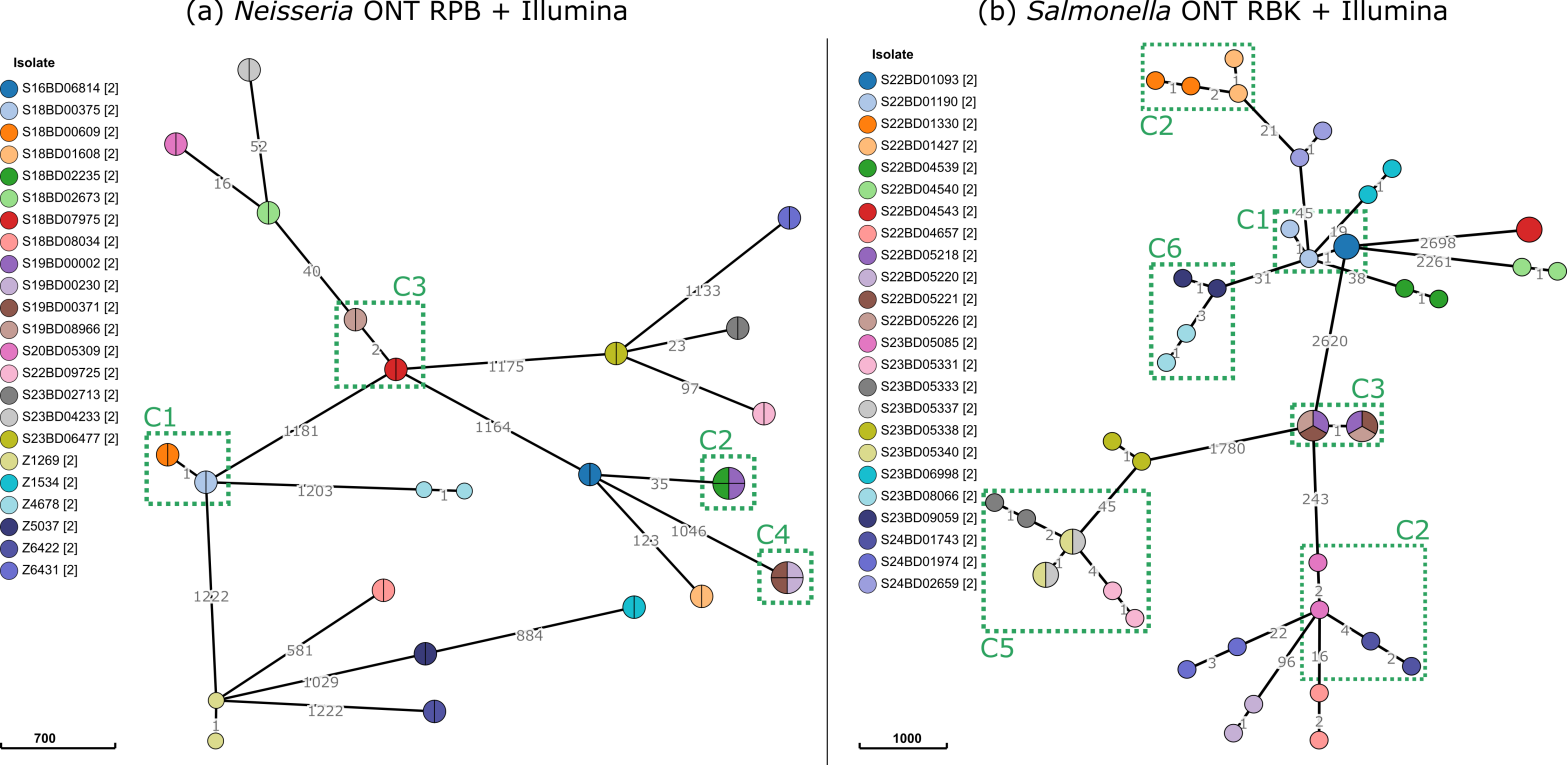
Minimum spanning tree for the unpolished *Neisseria* ONT RPB and unpolished *Salmonella* ONT RBK assemblies combined with the corresponding Illumina datasets. This plot shows the minimum spanning tree phylogeny combining (**a**) the ONT R10 RPB and the Illumina data sets as a network for the *Neisseria* isolates and (**b**) the ONT R10 RBK and Illumina data sets for the *Salmonella* isolates. Branch lengths and the scale bar correspond to the number of cgMLST allele differences and are scaled logarithmically. Nodes are colored by isolates, and clusters are indicated with green boxes. Note that for all *Neisseria* isolates, replicate data sets for the same sample using either ONT or Illumina, clustered at distance zero, except for isolates Z1269 and Z4678, which had a pairwise distance of one allele. For *Salmonella*, the branch lengths between replicate data sets for the same sample using either ONT or Illumina were slightly larger, but all clustered isolates matched the clustering in the reference phylogeny, separated from other isolates. A similar figure for the *Neisseria* RBK and *Salmonella* RPB datasets is provided in Fig. S20. The visualizations were created using GrapeTree [5].

To evaluate the combination of Illumina and ONT data separately into the same phylogenomic analysis, MSTs were constructed combining data sets generated by both methods (e.g., the *Neisseria* RPB-only and Illumina-only data sets in Fig. 4). The pairwise distances between the ONT-only and corresponding Illumina data sets are shown in Fig. S12 and S13 for *Neisseria* and *Salmonella*, respectively. The proportion of pairwise cgMLST allele mismatches between replicates was highest for the *Neisseria* RBK data sets, almost entirely due to alleles that were not called as perfect hits in the ONT data sets. For the *Neisseria* RPB data sets, the number of pairwise differences between the alleles called in the replicates generated with the ONT-only and Illumina-only data ranged from zero to eight, with a median of zero. The majority of these mismatches were due to alleles that were not called in the Illumina data. Interestingly, the four Illumina data sets with a relatively high number of these missing alleles also had the lowest N50 values, suggesting that the missing alleles may be a consequence of lower assembly quality (i.e., more fragmented) rather than being different allele calls. For both the unpolished and polished RPB assemblies, the total number of “true” mismatches (i.e., a different allele called as a perfect hit) across all data sets was two. Note that since the MSTreeV2 algorithm does not consider missing alleles as mismatches, the replicates (i.e., the same isolate sequenced with a different method) of 22 of the 24 *Neisseria* isolates clustered at distance zero in the corresponding phylogeny (Fig. 4). Using the definitions shown in Fig. 1 to determine clustering performance, an accuracy of 100% was obtained. For the *Salmonella* data sets generated with the RBK kit, the number of mismatches ranged from zero to six, with a roughly equal distribution of missing alleles in the ONT and Illumina data. After polishing, the maximum number of mismatches decreased to three alleles. In contrast to the *Neisseria* phylogeny, there were many more replicates that did not cluster at distance zero in the minimum spanning tree, consistent with the higher number of “true” mismatches (i.e., mismatches other than missing loci). For the *Salmonella* data sets generated with the RPB kit, the maximum number of mismatches in the unpolished and polished assemblies was three and two, respectively. Notwithstanding, the overall clustering performance for these data sets was also 100%.

### Investigation of mismatches and methylation calling

An overview of the types of mismatches between the hybrid assemblies and unpolished ONT-only assemblies in the cgMLST loci is provided in Table 5. For *Neisseria*, the only mismatch within the cgMLST loci in the unpolished RPB assemblies compared to the hybrid assemblies was an indel in sample Z4678 (Fig. S14). Note that there was an additional cgMLST mismatch (Fig. 3) when comparing the unpolished ONT-only S16BD06814 assembly with the hybrid assembly. The origin of this mismatch was not investigated, as this locus was not called as a perfect hit in the S16BD06814 hybrid assembly, which was used to determine the genomic location of the cgMLST loci. For the RBK kit, 311 mismatches were found across all high-quality unpolished ONT-only assemblies, with the majority being SNPs (*n* = 208). Interestingly, 199 of these 208 SNPs occurred at G or C positions in the hybrid assembly, which were modified into A and T, respectively (i.e., the same mutation on opposite strands). Of the 208 discrepant SNPs, 154 (77.39%) were located at sites called as methylated, the vast majority being 5mC (*n* = 152), with two instances of 6mA (Fig. S15). For 21 of the remaining 54 mismatches at positions not reported as methylated, methylation was detected in the next base upstream and/or downstream for the following: 6mA (*n* = 11), 5mC (*n* = 9), and both (*n* = 1). The extremely high proportion of the C/G to A/T mismatch compared to others, even when positions with detected methylation are discarded, and the absence of this mismatch in the RPB assemblies may indicate that the number of mismatches associated with methylation is underestimated. A visualization of an alignment at a methylated position resulting in a cgMLST mismatch is shown in Fig. S16. For the majority of the 103 erroneous indels, methylation was detected at the position of the indel (*n* = 33) or one base up/downstream (*n* = 44). Only eight indels were located in homopolymer regions of six bases or more (Fig. S17), for which no methylation was detected, including in the upstream and downstream bases. The remaining 18 indels were not located in homopolymer regions, and no methylation was detected at the corresponding site or one base upstream or downstream.

**TABLE 5:** Overview of the mismatches between the hybrid assemblies and unpolished ONT-only assemblies in the cgMLST loci^*a*^

	*Neisseria*		*Salmonella*	
	RBK	RPB	RBK	RPB
No. of isolates	23	23	23	23
SNPs				
Methylated	154	0	0	0
Methylated (±1 base)	21	0	6	0
Unmethylated	33	0	5	0
Total	208	0	11	0
Indels				
Methylated	33	0	0	0
Methylated (±1 base)	44	0	0	0
Homopolymer	8	1	28	28
Non-methylated and non-homopolymer	18	0	0	0
Total	103	1	28	28

^*a*^ Mismatches were identified using dnadiff. Only cgMLST loci detected as a perfect hit in the corresponding hybrid assemblies were considered. Abbreviations: single nucleotide polymorphisms (SNPs). Note that the *Neisseria* S18BD08604 isolate and the *Salmonella* S23BD06998 isolate were omitted from this analysis because of low-quality RBK data sets.

For *Salmonella*, the number of SNPs and indels within the cgMLST loci was similar for both barcoding kits, with 39 and 28 mismatches for the RBK and RPB kits, respectively (Fig. S18). For the RBK kit, the majority of mismatches were indels (71.79%), all but one of which were located in homopolymers of length six or longer (Fig. S17). An example of a homopolymer-related error leading to a mismatched cgMLST allele call is provided in Fig. S19. All SNPs were C/G to T/A mutations (*n* = 11), but, in contrast to *Neisseria*, methylation was not detected at any of these positions. However, for six SNPs, 6mA methylation was detected one base up- or downstream. For the RPB kit, all mismatches were indels, and no SNPs were found. All mismatches were located within homopolymer regions of size six bp or longer. Interestingly, the ONT data contained one missed base compared to the hybrid assembly for all indels within cgMLST loci for both species, except for a single indel in the STMMW_21691 locus in the *Salmonella* S22BD01190 RBK data set. Although the same number of erroneous indels were detected in the RPB and RBK data sets, these were not the same. However, certain loci, such as STMMW_17581 (i.e., 27 mismatches across all data sets) and STMMW_27051 (i.e., eight mismatches across all data sets), were commonly associated with homopolymer-related errors for both barcoding kits.

## DISCUSSION

In this study, we have evaluated ONT R10 sequencing for cgMLST-based allele calling and clustering for bacterial outbreak investigation on a selection of *Neisseria* and *Salmonella* isolates. The performance of the RBK and RPB barcoding kits was assessed both with and without long-read polishing.

Overall, cgMLST allele calling on the ONT R10 data sets was very accurate, with over 99% of alleles called correctly, with the exception of the *Neisseria* data sets sequenced using the RBK kit. In these data sets, methylation-related errors had a substantial negative impact on the accuracy of cgMLST allele calling and subsequent clustering. A relatively large number of SNPs (*n* = 208) were identified between the unpolished ONT-only assemblies and the hybrid assemblies, with 74.04% of these located at positions that were identified as methylated. An additional 10.10% of these mismatches were located one base upstream or downstream of a methylated position (Table 5). Our results also suggest that methylation may cause indel errors, as 74.76% of the erroneous indels between the hybrid and unpolished ONT-only assemblies were located at methylated sites, or one base up/downstream (Fig. S15). Interestingly, the frequency of methylation-related errors was strongly strain-dependent, as seen elsewhere (16, 17) (Fig. 3). These strain-dependent differences may be caused by differences in the methylation profiles of the strains. For example, some hypervirulent *Neisseria meningitidis* strains have been shown to carry different methyltransferases that can regulate gene expression (42). Similarly, methylation profiles have been shown to vary widely between bacteria (43). The overall proportion of errors in the *Neisseria* RBK data sets was substantially higher than for the *Salmonella* RBK data sets, where six out of a total of eleven SNP mismatches in the unpolished ONT-only assemblies were located at positions called as methylated. Interestingly, in contrast to the *Neisseria* RBK data sets, none of the erroneous indels were associated with methylation. In the *Salmonella* data sets, all methylation-related mismatches were attributed to 6mA methylation, whereas in *Neisseria*, almost all were caused by 5mC methylation. Note that because the screening was limited to three types of methylation, other types of methylation that could affect the accuracy of base calling may have been missed. The PCR-based RPB kit removes methylation prior to sequencing (16) and resulted in near-perfect concordance with the hybrid assemblies for both *Neisseria* and *Salmonella*. An additional potential advantage of this kit was the reduced variation in sequencing depth of isolates within the same run. Consequently, the likelihood of having to re-sequence low-depth isolates is likely lower. However, the nature of this kit results in shorter read lengths, leading to more fragmented assemblies (Fig. S3). Therefore, for applications where obtaining near-complete genomes is required, the use of the RPB kit could limit the added value of long-read ONT sequencing. In addition, the protocol is more laborious and time-consuming. Given the substantial variation in cgMLST allele calling accuracy across the species and strains tested, we recommend evaluating different barcoding kits to identify the most appropriate one for the intended application. Performance can be assessed by comparing allele calls with Illumina data, as these are not affected by epigenetic modifications and ONT-specific homopolymer errors. However, comparing to hybrid assemblies may be preferable, as these assemblies tend to be more complete and accurate. For example, we found several typing loci were not adequately covered by the Illumina data, especially for *Neisseria*.

As an alternative to the RPB kit, polishing with medaka was tested to resolve potential methylation-related and other errors (44, 45). Overall, polishing slightly reduced the number of mismatched cgMLST loci. However, polishing also frequently introduced novel errors. The second most common source of errors was homopolymers, a well-known phenomenon in nanopore-based sequencing (46). These errors were observed for both kits (Fig. S17). However, the impact was relatively limited, especially when compared to the overall level of error observed in the *Neisseria* RBK data sets. Future advances in polishing, such as the recently released v2 of the Medaka polishing software and updated models, may help to better correct both types of error in the future.

The proportion of cgMLST loci that could be identified as perfect hits was considerably lower for *Salmonella* than for *Neisseria*, irrespective of sequencing technology and protocol (Table 2). The main cause of uncalled alleles in the *Salmonella* data sets was multi-hits, shown schematically in Fig. S7. This issue stems from database curation. Ideally, overlapping alleles should be avoided, as they can lead to a loss of resolution or, in the worst case, mismatches when comparing strains. In this study, we used the third iteration of the *Neisseria* cgMLST scheme, which has been refined over time by removing problematic loci. When using the first iteration of the cgMLST scheme, we also observed a reduced proportion of perfect hits (Table S3). These findings highlight how the choice and curation of the cgMLST scheme can affect allele calling and subsequent clustering results.

Despite these limitations, ONT R10 sequencing enabled accurate cgMLST-based clustering of both species, consistent with findings reported for other species (13, 15). For *Salmonella*, both the RBK and RPB kits produced phylogenies that were very similar to the results obtained with hybrid sequencing data, leading to identical cluster interpretations using the loose cluster definition threshold. When using the strict allele threshold for cluster membership, the clustering performance was slightly reduced for the unpolished RBK and the polished RPB data sets due to a few isolates that were misclassified (Table 3). In these cases, the isolates were already at a pairwise distance close to the threshold in the reference phylogeny, and discrepancies in a few loci caused the pairwise distance to exceed the threshold. It may, therefore, be beneficial to apply slightly more lenient thresholds when integrating data from multiple sequencing technologies to account for potential technology-specific discrepancies, followed by additional investigation of detected clusters. The number of cgMLST mismatches observed in this study for *Salmonella* was lower than the number of mismatches reported in previous studies. Xian et al. previously reported ~16 cgMLST mismatches per strain using ONT R9 sequencing, which could be reduced to ~5 using a database-based homopolymer correction approach (47). Hong et al. similarly evaluated ONT R10 sequencing using the ligation and the rapid barcoding kit, and the number of average mismatches per strain ranged from 7 to 9, depending on the barcoding kit (48). In the current study, we have observed fewer mismatches, likely due to improvements in the sequencing technology and basecalling model, as Xian et al. used an older chemistry and Hong et al. used v4 of the SUP model, compared to v5 used in this study. In addition, by sequencing with a PCR-based barcoding kit, we were able to demonstrate a link between methylation and sequencing errors that had not been observed in these previous studies. For *Neisseria*, methylation-related errors substantially impacted the results on the data sets generated with the RBK kit, leading to different clustering compared to the hybrid assemblies (Fig. S9 and S20; Table 3). The RPB kit did not suffer from this issue and led to nearly identical results as the hybrid assembly approach, with at most a single mismatched allele (Fig. 4) and identical clustering with both thresholds. To the best of our knowledge, this is the first study for *Neisseria meningitidis* to focus on the performance of cgMLST-based allele calling and clustering using ONT data. Based on the errors observed with the RBK kit, we currently advocate the use of the RPB kit for *Neisseria*, despite it being more laborious and time-consuming, although future updates of the sequencing chemistry, basecalling model, and/or polishing methods could potentially resolve such methylation-induced errors.

Given the extensive collection of cgMLST profiles generated by short-read sequencing accumulated by laboratories and public resources such as PubMLST and EnteroBase (3, 4), we also evaluated the performance when combining cgMLST results obtained with ONT and Illumina sequencing. This could be crucial, for example, to link isolates sequenced with ONT to historical clusters or to combine data from laboratories using different sequencing technologies. Our results showed that the pairwise allele distances between replicates of the same sample sequenced with different technologies were relatively small, regardless of the sequencing method and/or polishing, except for the *Neisseria* ONT data sets generated with the RBK kit (Fig. S12 to S14). However, as clusters are usually defined using allele thresholds of a relatively small number of alleles (4), even minor discrepancies due to the employed sequencing technology can impact clustering. This was observed in the evaluation of the clustering performance, which was perfect for the loose threshold, but was lower for the strict threshold (Table 3). It should be noted that the method used to extract clusters can also have a major impact on the results obtained. For example, in the MSTreeV2 tree-building algorithm, missing alleles do not contribute to the difference between strains (5). Consequently, 22 out of 24 *Neisseria* replicates sequenced using the ONT RPB kit and Illumina clustered at distance zero (Fig. 4a), as almost all mismatches were due to alleles not called in one of the data sets (Fig. S12). Similar to allele calling, an evaluation of suitable cluster definition thresholds for the intended application is hence critical (49, 50). In summary, our results show that Illumina and ONT sequencing can be used interchangeably for cgMLST allele calling and cgMLST-based phylogenetic investigation, provided methylation-induced errors are accounted for, and data processing and cluster definitions are adjusted accordingly.

While our study highlights some critical aspects and considerations for ONT R10-based cgMLST analysis for bacterial outbreak analysis, there are several limitations. First, only a single allele calling method was evaluated while numerous alternatives are available (51–53). The allele calling method could affect performance, with some methods being more or less affected by the methylation- and homopolymer errors observed here. In addition, the assembly and polishing methods may also affect performance. Notwithstanding, our employed bioinformatics approach corresponds to standard practice for cgMLST allele calling using ONT data. Second, our evaluation was limited to two species, and the application of this approach to other species will require further evaluation, as we observed substantial interspecies variation but also intraspecies variation within *Neisseria*. Different species will need to be evaluated separately to assess the potential impact of methylation-related errors, homopolymer-related errors, or other potential sources of error that were not observed in our *Neisseria* or *Salmonella* data sets. We also focused on including data from well-described outbreaks, corresponding only to a relatively small number of STs. However, this only covers a small fraction of the diversity of both species, given the huge number of known STs. Third, the performance on cgMLST allele calling does not necessarily transfer to other genomic assays, such as AMR or virulence gene detection, *in silico* serotyping, plasmid reconstruction, etc. Further studies with high-quality phenotypic or genomic reference data are required to evaluate the performance of other bioinformatics assays using ONT sequencing. Fourth, there were no replicates for data sets generated using the same protocol, as only a single ONT flow cell was used for all barcoding kits and species combinations, while there still existed considerable variation in active pores, throughput, etc. Consequently, for the integration of ONT sequencing for cgMLST-based cluster detection into the routine activities of clinical and public health laboratories operating under a quality system and requiring certification and/or accreditation, future large-scale validation is still required to assess the performance and stability of ONT R10 sequencing.

In conclusion, our results show that ONT R10 sequencing is well-suited for core-genome MLST-based phylogenomic analysis, taking into account species-specific differences, but library preparation protocols should be adapted to cope with erroneous base calls due to DNA methylation and other ONT-specific errors. As ONT-based sequencing is still rapidly evolving, advances in sequencing chemistry, base calling models, and analysis software can still improve results, address potential issues, and achieve even greater accuracy in cgMLST-based allele calling and clustering. The methodology presented in this paper could serve as a baseline for other laboratories to incorporate ONT sequencing for cgMLST analysis and connect their data with historical cgMLST data sets generated using Illumina sequencing.

## Data Availability

The data sets supporting the conclusions of this study have been deposited in the NCBI SRA, and the corresponding BioSample accession numbers are provided in Table S5. The raw ONT signal data are available in SquidBase at https://squidbase.org/submissions/SQB000006 (RBK) and https://squidbase.org/submissions/SQB000007 (RPB).

## SUPPLEMENTAL MATERIAL

Supplemental material jcm.00410-25-s0001.docx Supplemental methods and results, Fig. S1 to S20, and Tables S1 to S7.

ASM does not own the copyrights to Supplemental Material that may be linked to, or accessed through, an article. The authors have granted ASM a non-exclusive, world-wide license to publish the Supplemental Material files. Please contact the corresponding author directly for reuse.
