# Supplementary material for: Oxford Nanopore Technologies R10 sequencing enables accurate cgMLST-based bacterial outbreak investigation of Neisseria meningitidis and Salmonella enterica when accounting for methylation-related errors
Source: J Clin Microbiol. 2025 Aug 22;63(10):e00410-25. doi: 10.1128/jcm.00410-25 (PMC12506013; doi:10.1128/jcm.00410-25)
Supplement: Supplemental material — Supplemental methods and results, Fig. S1 to S20, and Tables S1 to S7. [file jcm.00410-25-s0001.docx]

SUPPLEMENTARY MATERIAL

**Oxford Nanopore Technologies R10 sequencing enables accurate cgMLST-based bacterial outbreak investigation of *Neisseria meningitidis* and *Salmonella enterica* when accounting for methylation-related errors**

# Figures

## **Figure S1: Oxford Nanopore Technologies (ONT) read statistics before and after filtering**

1. ***Neisseria***


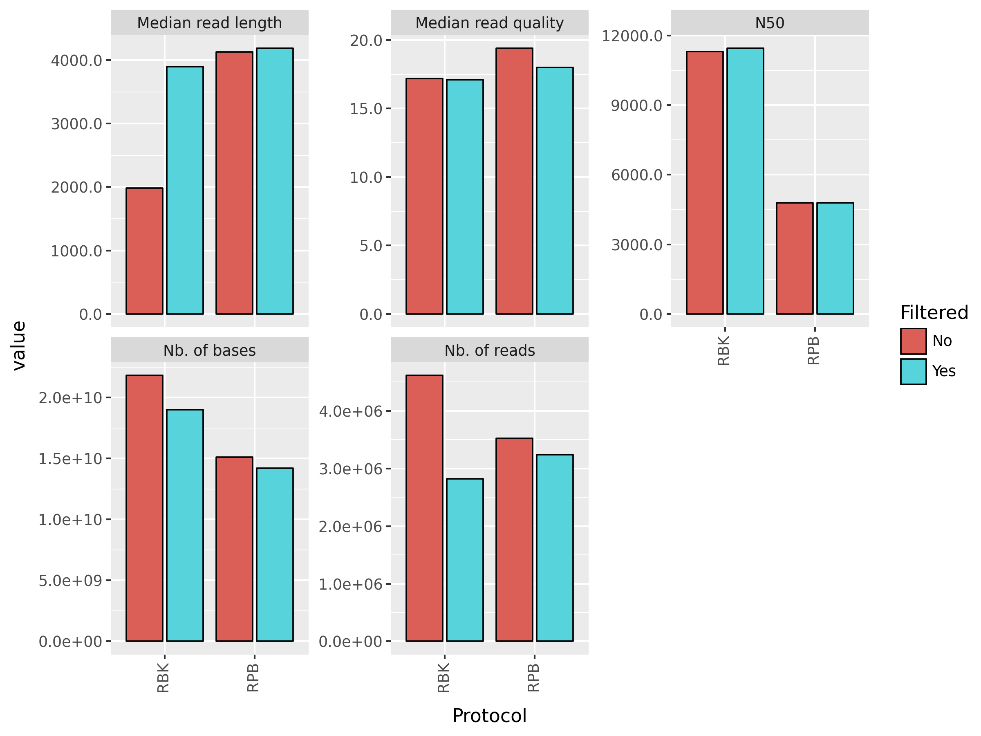


1. ***Salmonella***


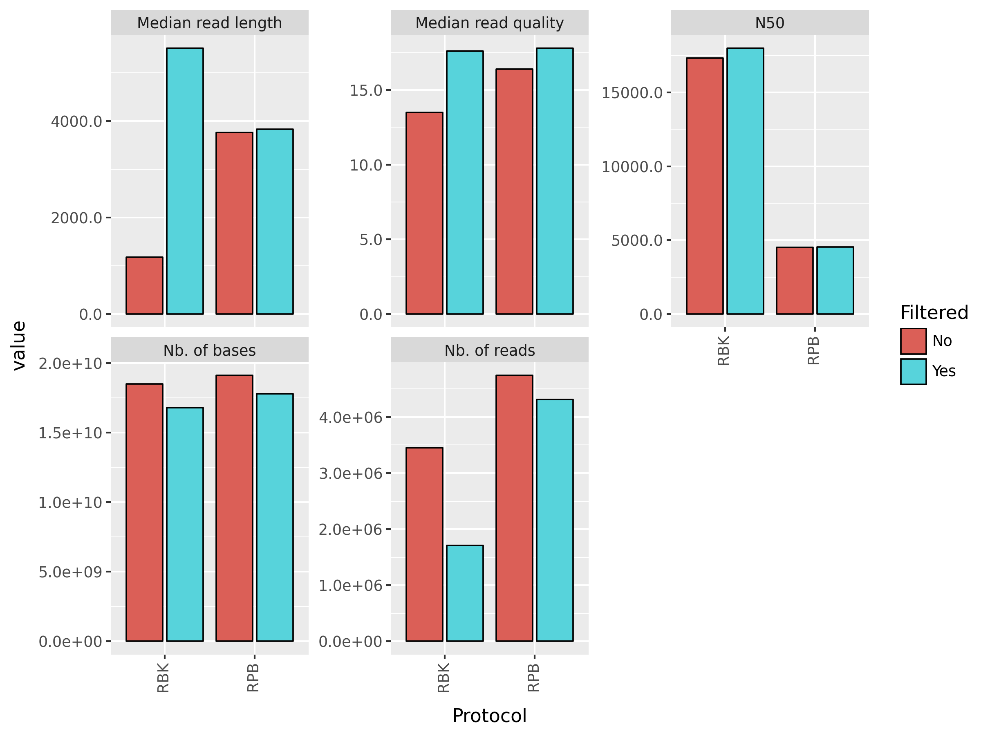


These plots show the run statistics for *Neisseria* (a) and *Salmonella* (b). The x-axis represents the barcoding protocol, the y-axis the value for the corresponding metric. The red and blue bars show the values before and after read filtering, respectively.

## **Figure S2: Read length and average read quality statistics for the *Neisseria* ONT runs**

1. **RBK**


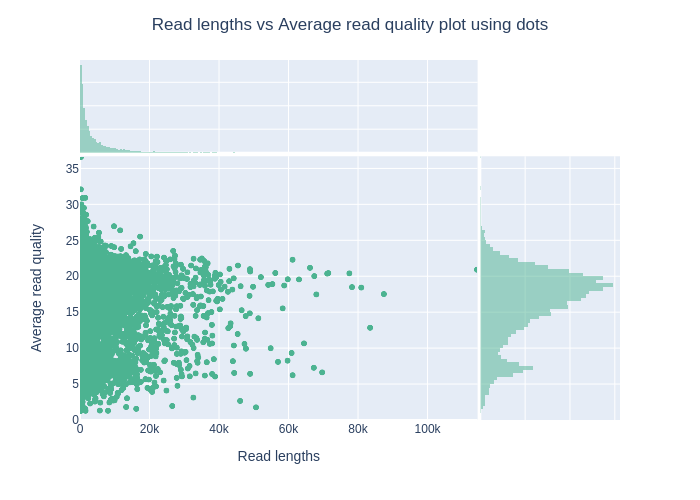


1. **RPB**


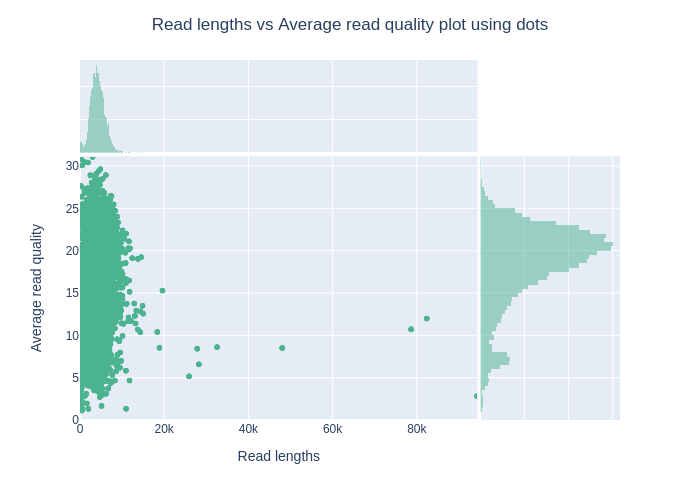


These plots show the read length and quality distribution for the *Neisseria* datasets generated with the RBK (a) and RPB (b) kit. The x-axis represents the read length and the y-axis represents the quality score. Each point corresponds to a single read. Plots were generated using NanoPlot [58].

## **Figure S3: Assembly metrics for the ONT datasets**

1. ***Neisseria***


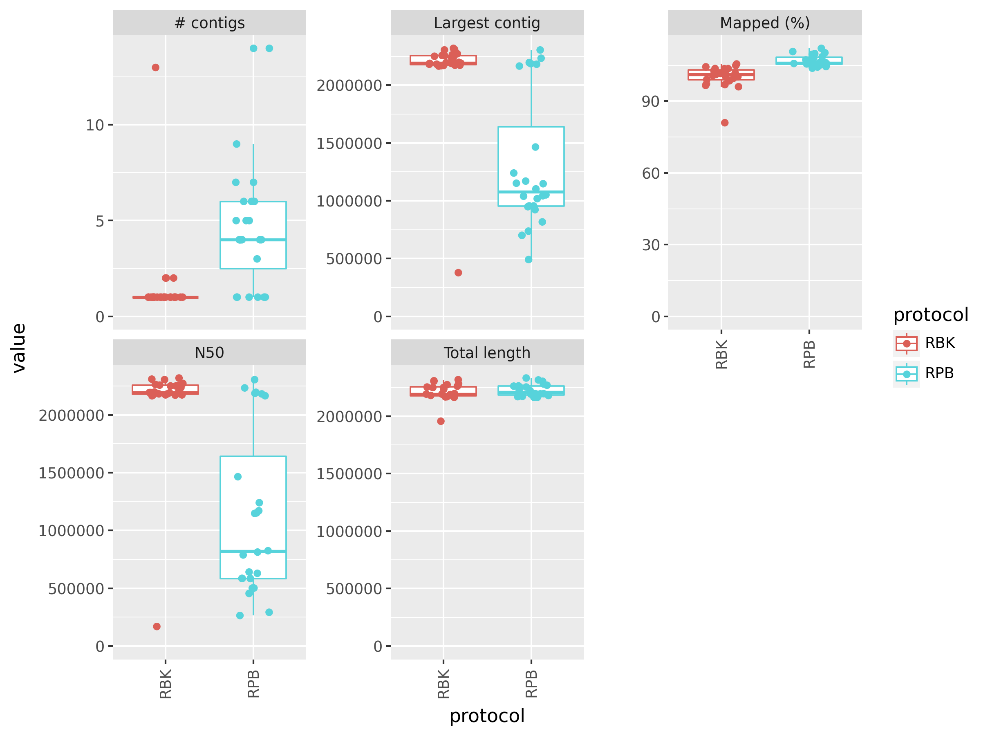


***b) Salmonella***


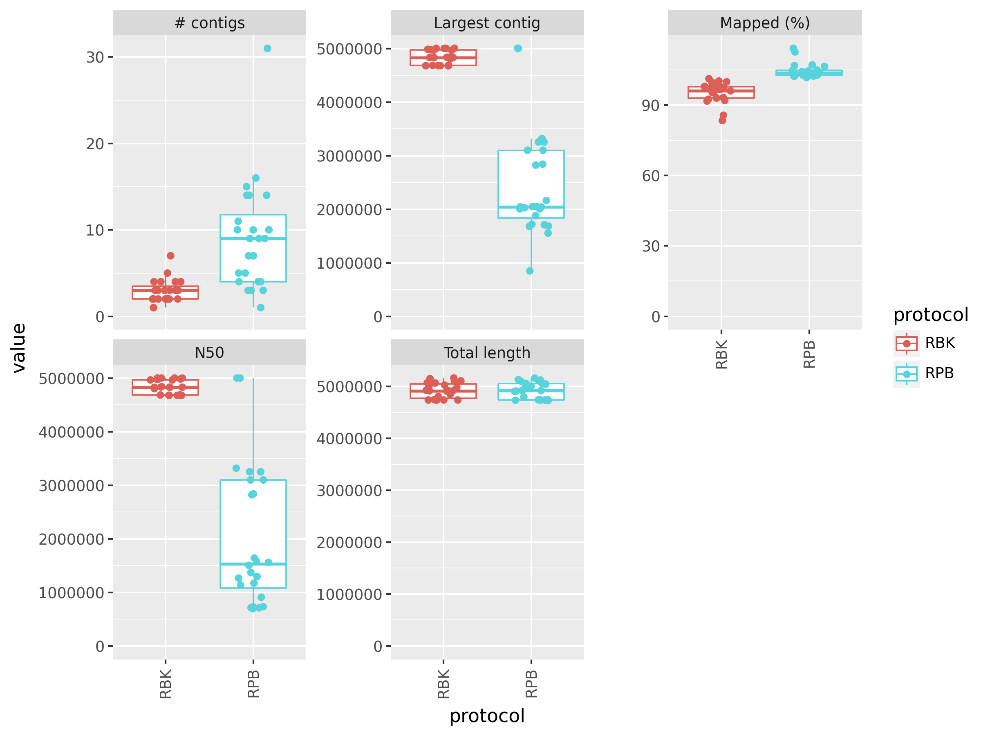


Assembly metrics for the ONT datasets generated with the RBK and RPB kits for *Neisseria* and *Salmonella*. Note that the *Salmonella* S23BD06998 RBK dataset was not included as it failed to assemble. The results for the polished assemblies have been omitted from these plots as they were almost identical to the unpolished assemblies.

## **Figure S4: Depth per isolate for the ONT datasets**


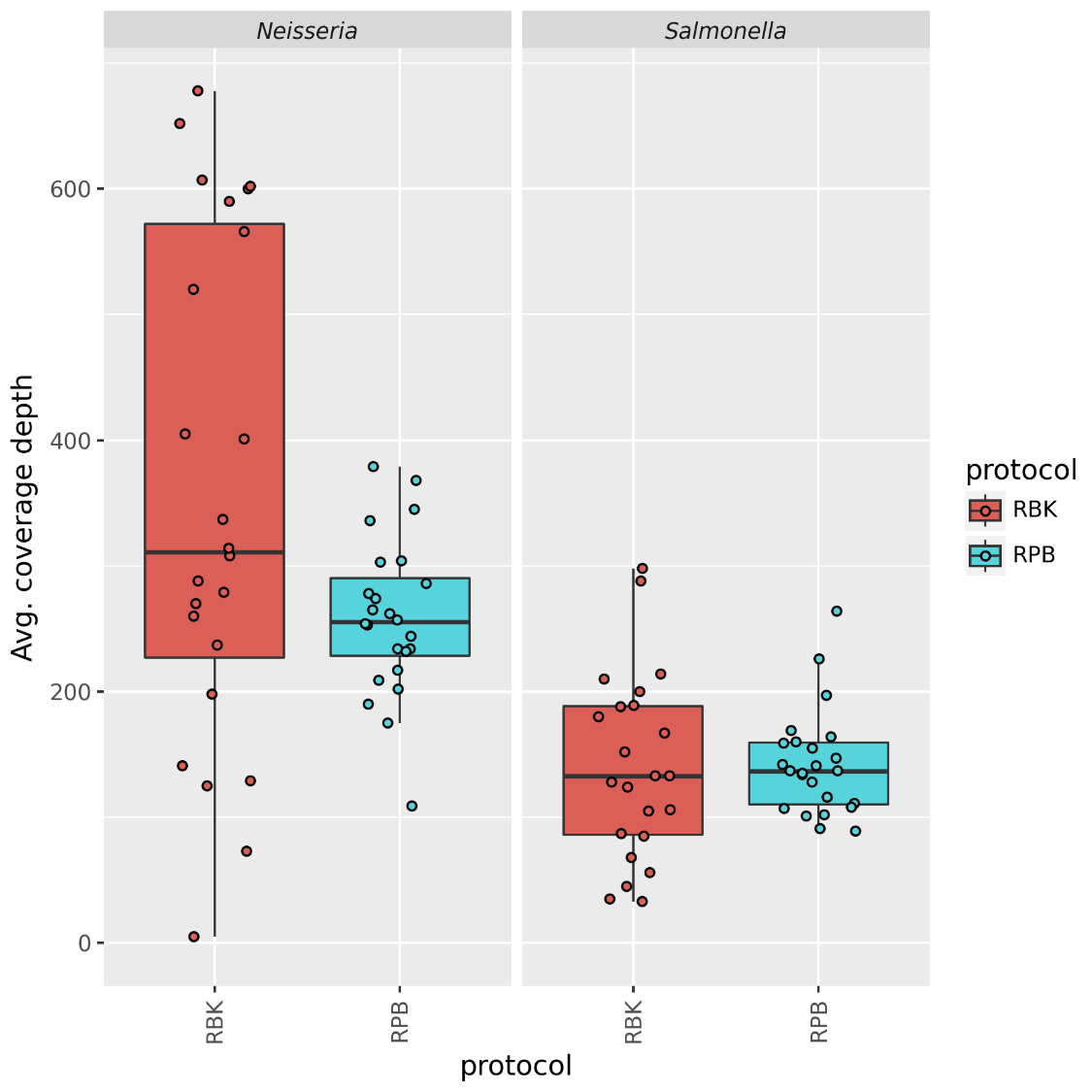


This plot shows the median sequencing depth for all isolates sequenced using the corresponding protocol as indicated on the x-axis. The results for *Neisseria* and *Salmonella* are shown on the left and right side, respectively. Note that the coverage was calculated by mapping against the assembled contigs, and that the *Salmonella* S23BD06998 RBK dataset failed to assemble and was therefore not included in this plot.

## **Figure S5: Assembly metrics for the Illumina datasets**

1. ***Neisseria***


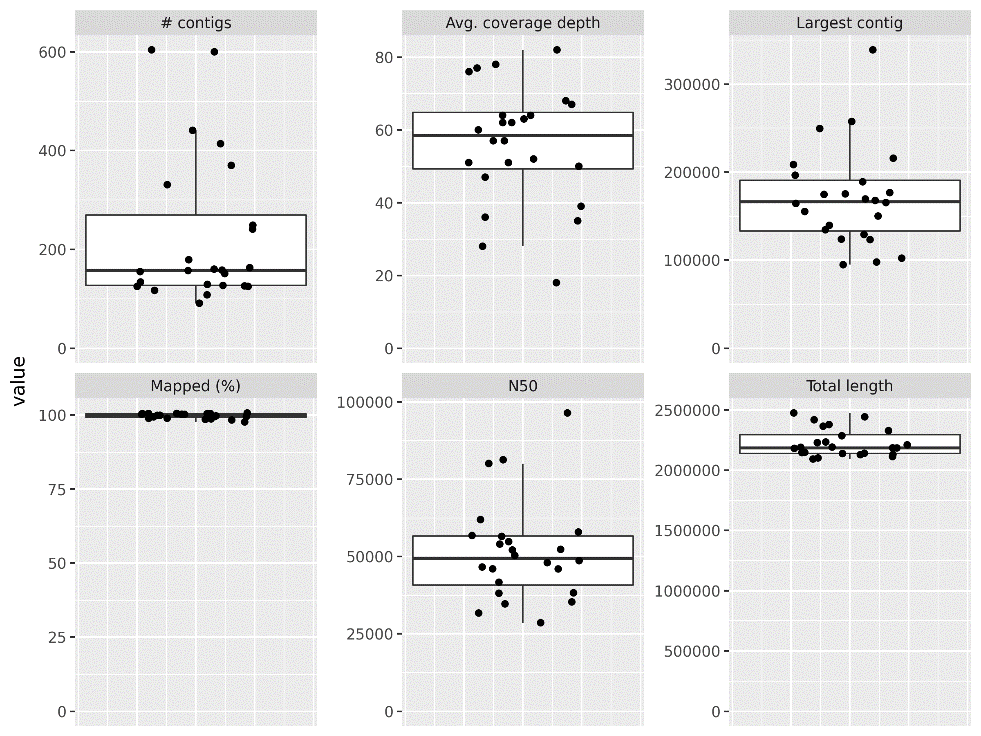


1. ***Salmonella***


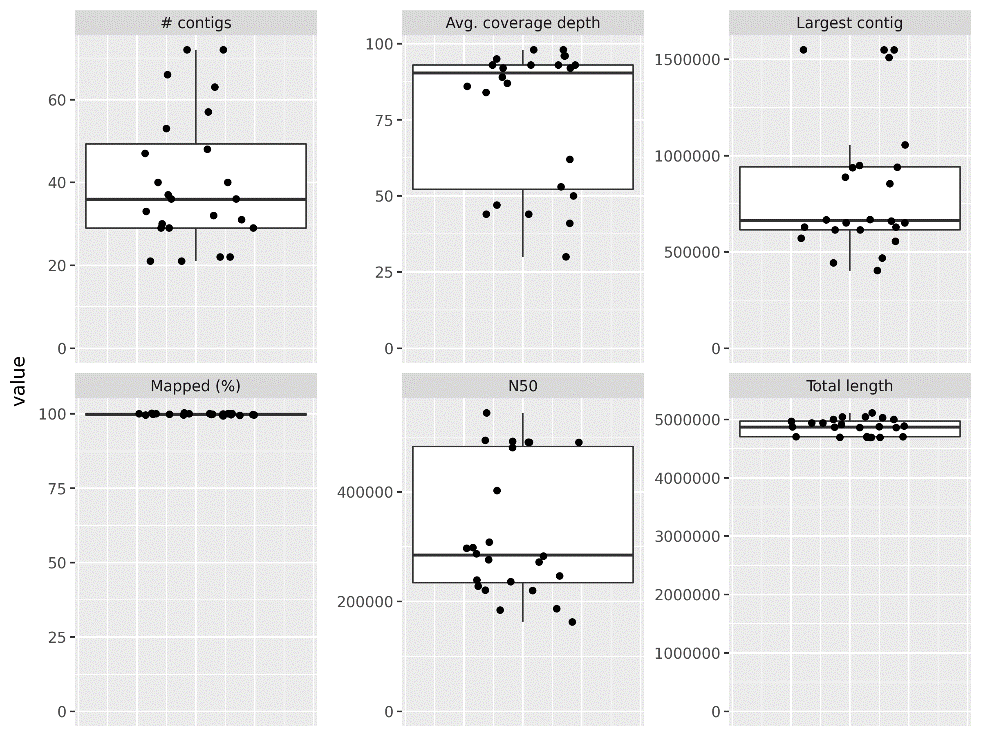


These plots show the assembly metrics for the Illumina datasets. Note that these metrics were calculated after downsampling of high coverage datasets.

## **Figure S6: Uncalled alleles in the hybrid assemblies and Illumina data**


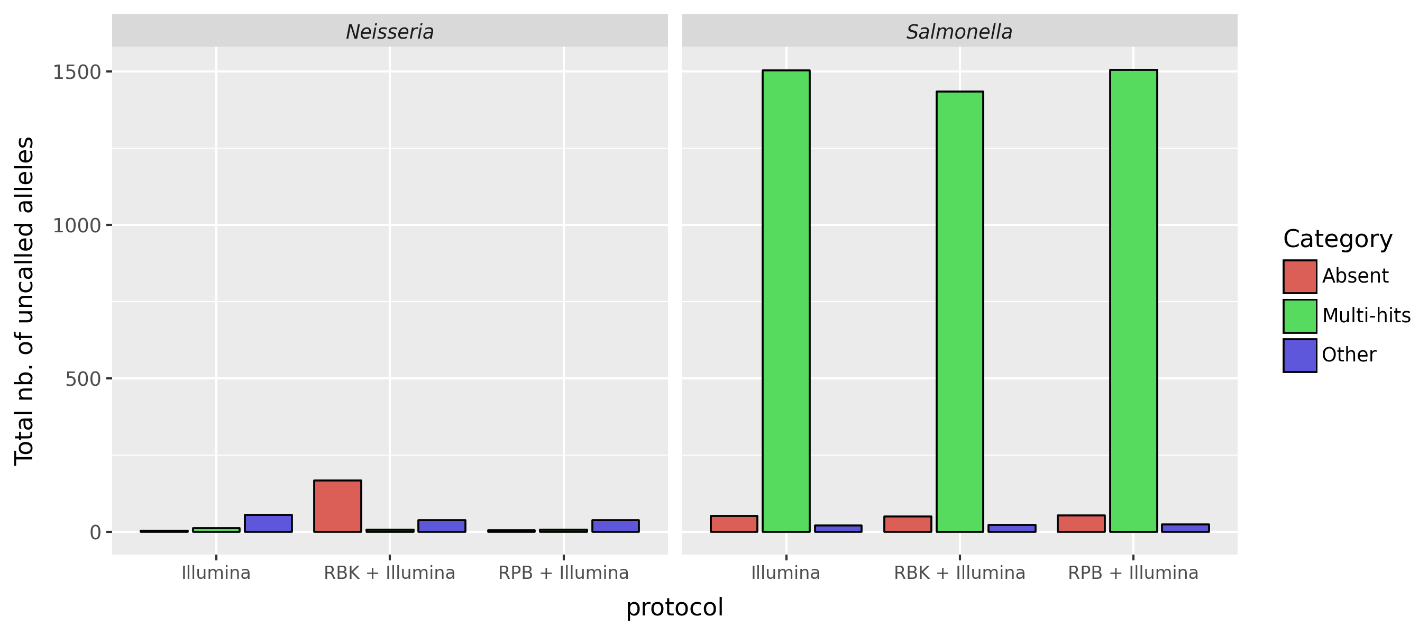


This visualization shows the total number of uncalled alleles in the hybrid and Illumina assemblies, summed over all datasets. The ‘absent’ category means that there are was no alignment that covered at least 60% of the locus with 90% sequence identity. The ‘multi-hits’ categories are cases where several alignments had the same score and no best hit could be selected. All other types of uncalled alleles are grouped in the ‘other’ category. RBK and RPB correspond to the hybrid assemblies generated from the RBK and RPB ONT reads combined with the corresponding Illumina data.

## **Figure S7: Schematic representation of a multi-hit**


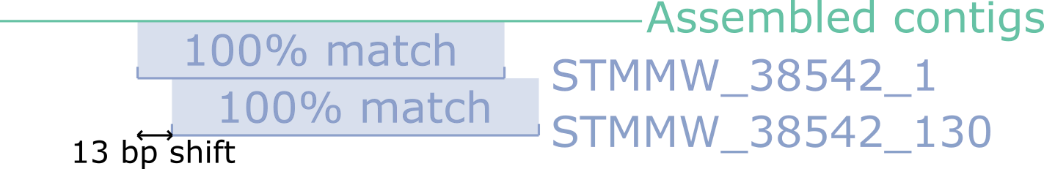


This figure shows a schematic representation of a multi-hit in the hybrid assembly of the *Salmonella* S22BD05226 RBK dataset. The sequences of the STMMW_38542_1 and STMMW_38542_130 alleles both match perfectly with the assembled contigs. The STMMW_38542_1 and STMMW_38542_130 alleles are both intact ORFs of exactly the same length (240 bp), with an identical sequence over the 227 overlapping base pairs. As a result, the pipeline is unable to select a best allele as the alignment statistics (including length) are identical for both alleles.

## **Figure S8: Reference phylogenies and clustering with the RBK datasets**

| ***Neisseria***  **RBK hybrid** | ***Salmonella***  **RBK hybrid** |
| --- | --- |
| 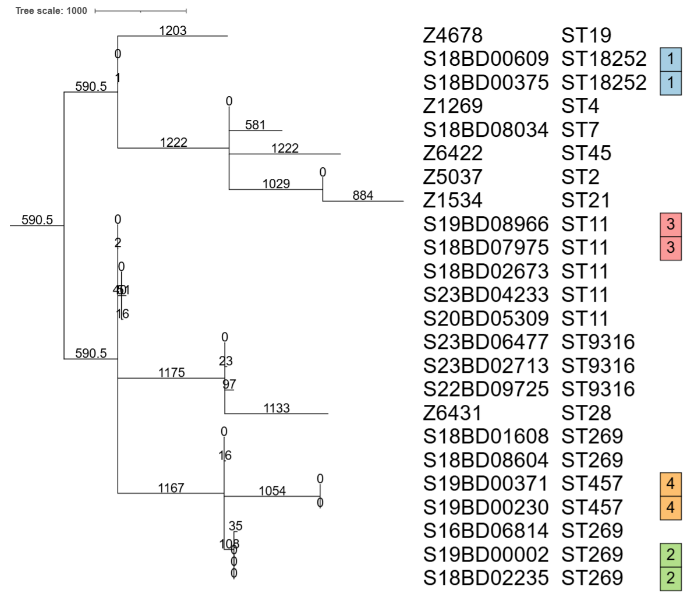 | 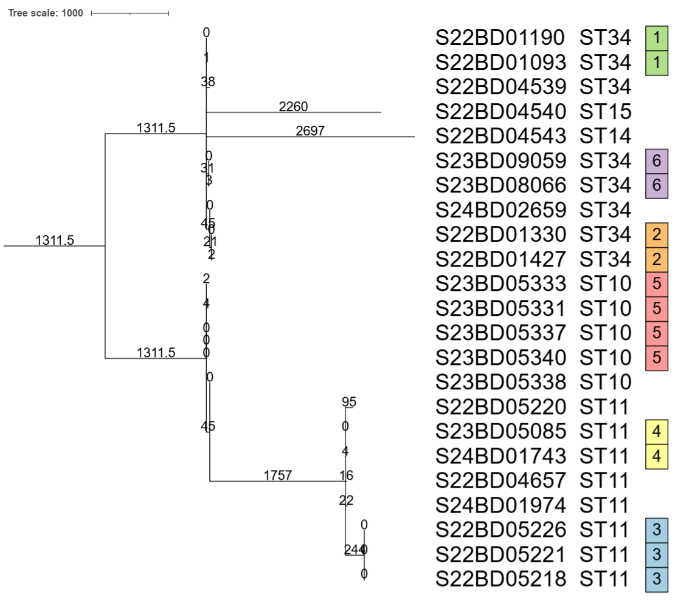 |

These plots show the reference MST phylogenies, displayed as cladograms, for the *Neisseria* and *Salmonella* datasets, constructed from the RBK hybrid assemblies. The annotations are from left to right: isolate name, sequence type (ST), and cluster membership. Clusters were defined as isolates that clustered within four and five alleles of each other for *Neisseria* and *Salmonella*, respectively (i.e., the strict thresholds). The loose thresholds are not shown as the obtained clusters were identical. The results for the RPB datasets are shown in Figure 2.

## **Figure S9: Pairwise allele distances for the clustered isolates in the *Neisseria* datasets**

1. **RPB**


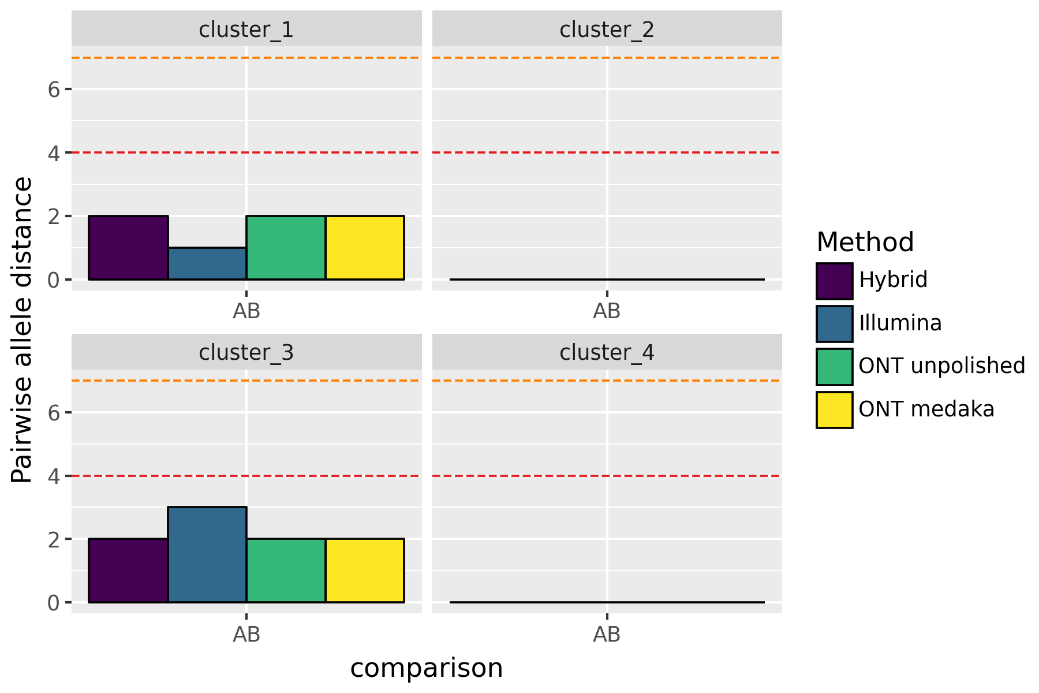


1. **RBK**


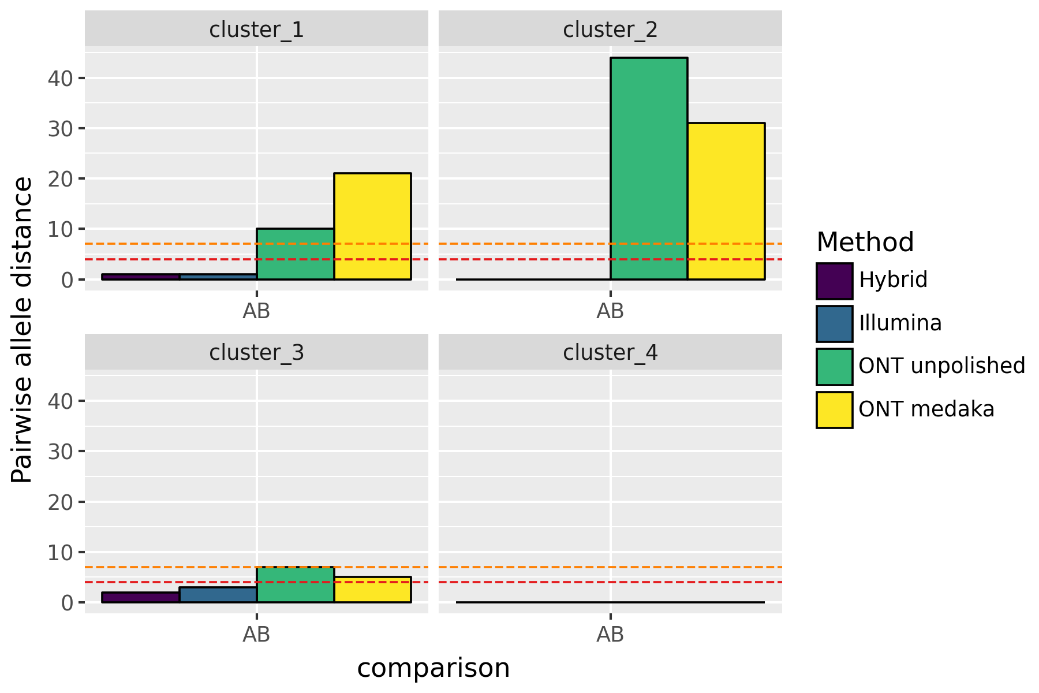


The plot shows the pairwise allele distances between the isolates that clustered together in the reference phylogeny. The results for the RPB (A) and RBK kit (B) are shown. The loose and strict clustering thresholds are shown as a horizontal red and orange lines, respectively. The clusters correspond to: (1) S18BD00375 (A), S18BD00609 (B); (2) S18BD02235 (A), S19BD00002 (B); (3) S18BD07975 (A), S19BD08966 (B); and (4) S19BD00230 (A), S19BD00371 (B).

## **Figure S10: Pairwise allele distances for the clustered isolates in the *Salmonella* datasets**

1. **RPB**


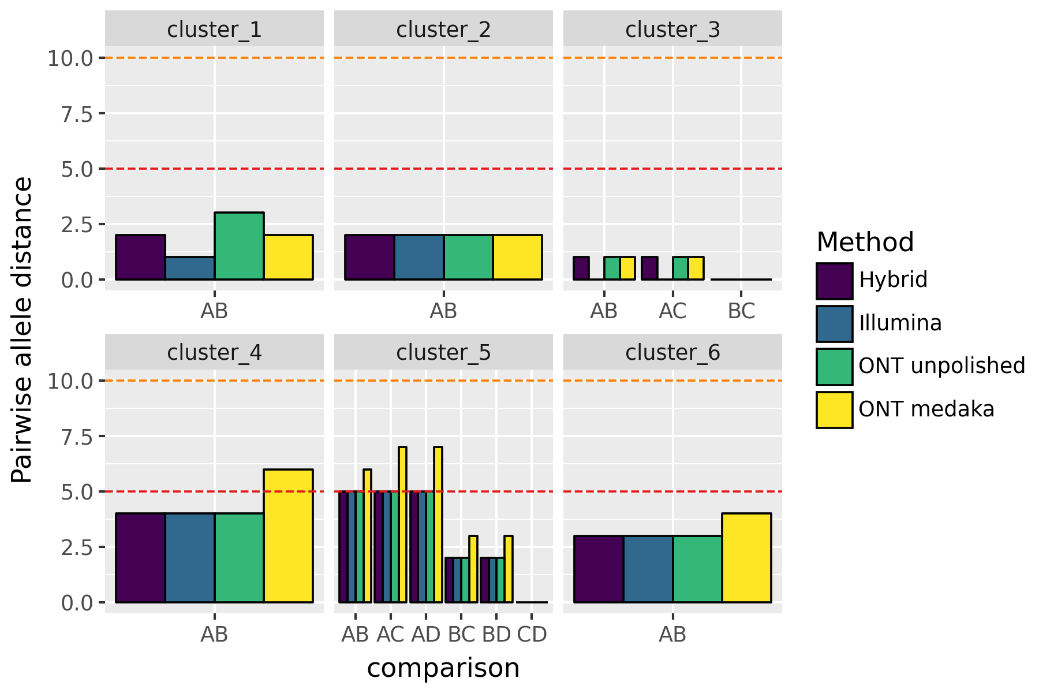


1. **RBK**


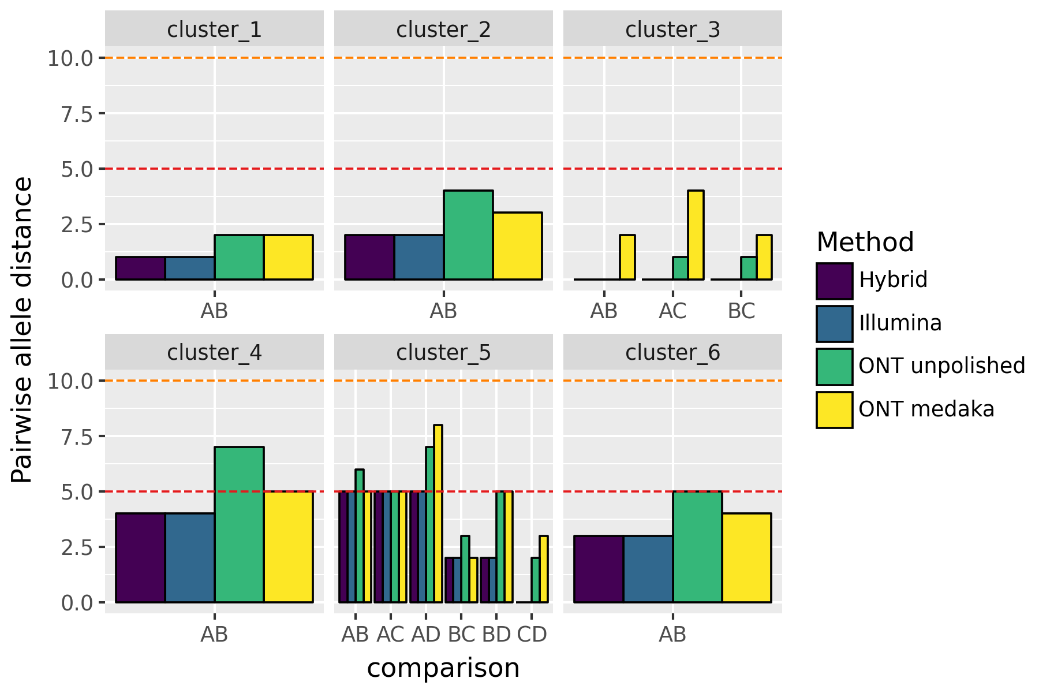


The plot shows the pairwise allele distances between the isolates that clustered together in the reference phylogeny. The results for the RPB (A) and RBK kit (B) are shown. The loose and strict clustering thresholds are shown as a horizontal red and orange lines, respectively. The clusters correspond to: (1) S22BD01093 (A), S22BD01190 (B); (2) S22BD01330 (A), S22BD01427 (B); (3) S22BD05218 (A), S22BD05221 (B), S22BD05226 (C); (4) S23BD05085 (A), S24BD01743 (B); (5) S23BD05331 (A), S23BD05333 (B), S23BD05337 (C), S23BD05340 (D); (6) S23BD08066 (A), S23BD09059 (B).

## **Figure S11: Mismatched cgMLST loci in the ONT-only assemblies compared to the hybrid assembly (grouped by locus)**


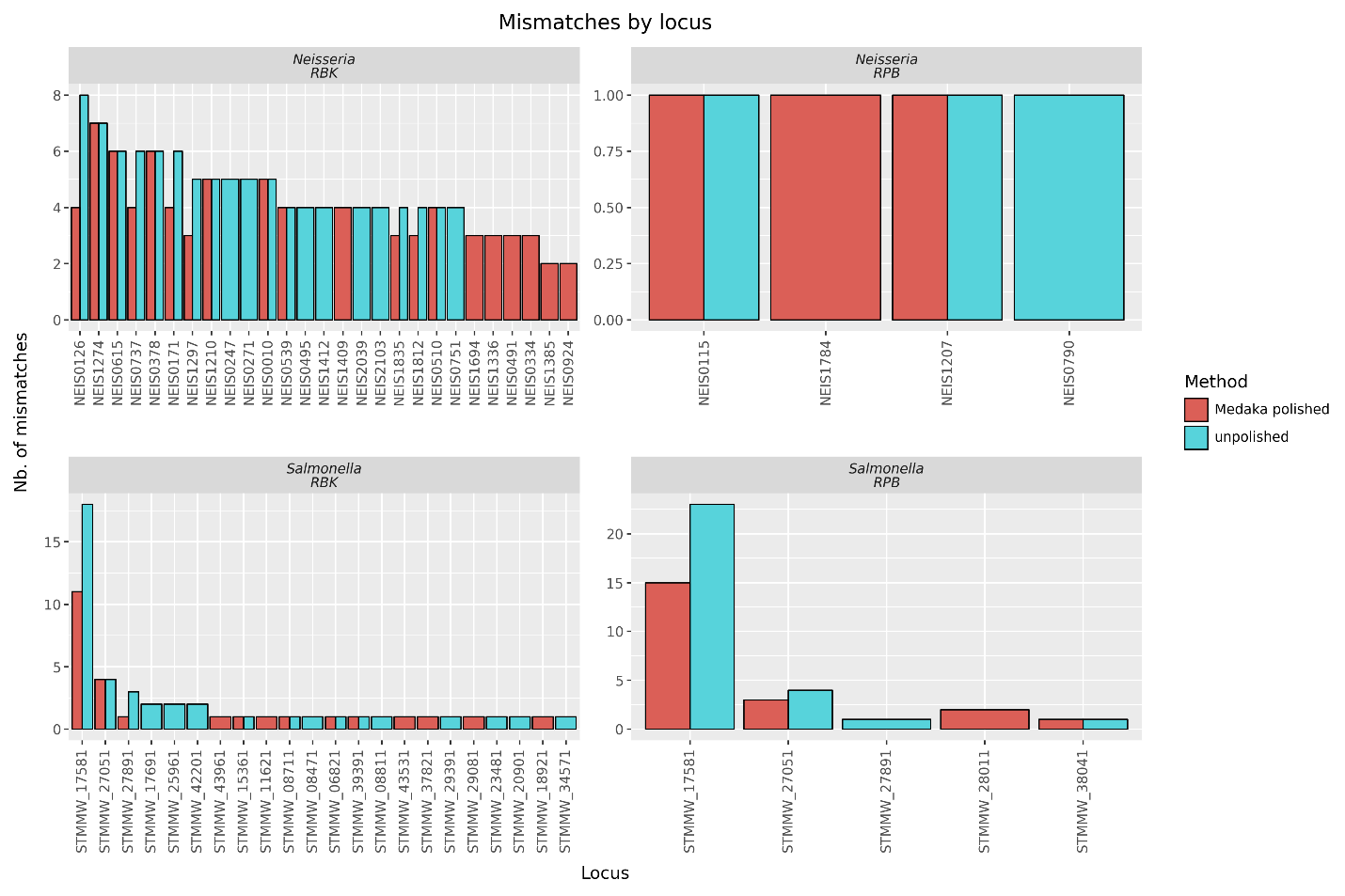


This figure shows the number of mismatches (y-axis) grouped by locus (x-axis). The bars are colored according to the assembly method: unpolished and polished (‘medaka’). Note that for the *Neisseria* RBK datasets only the first 20 out of 333 loci with mismatches are shown for visual clarity.

## **Figure S12: Mismatched cgMLST loci in the ONT-only datasets compared to Illumina for the *Neisseria* datasets**

***
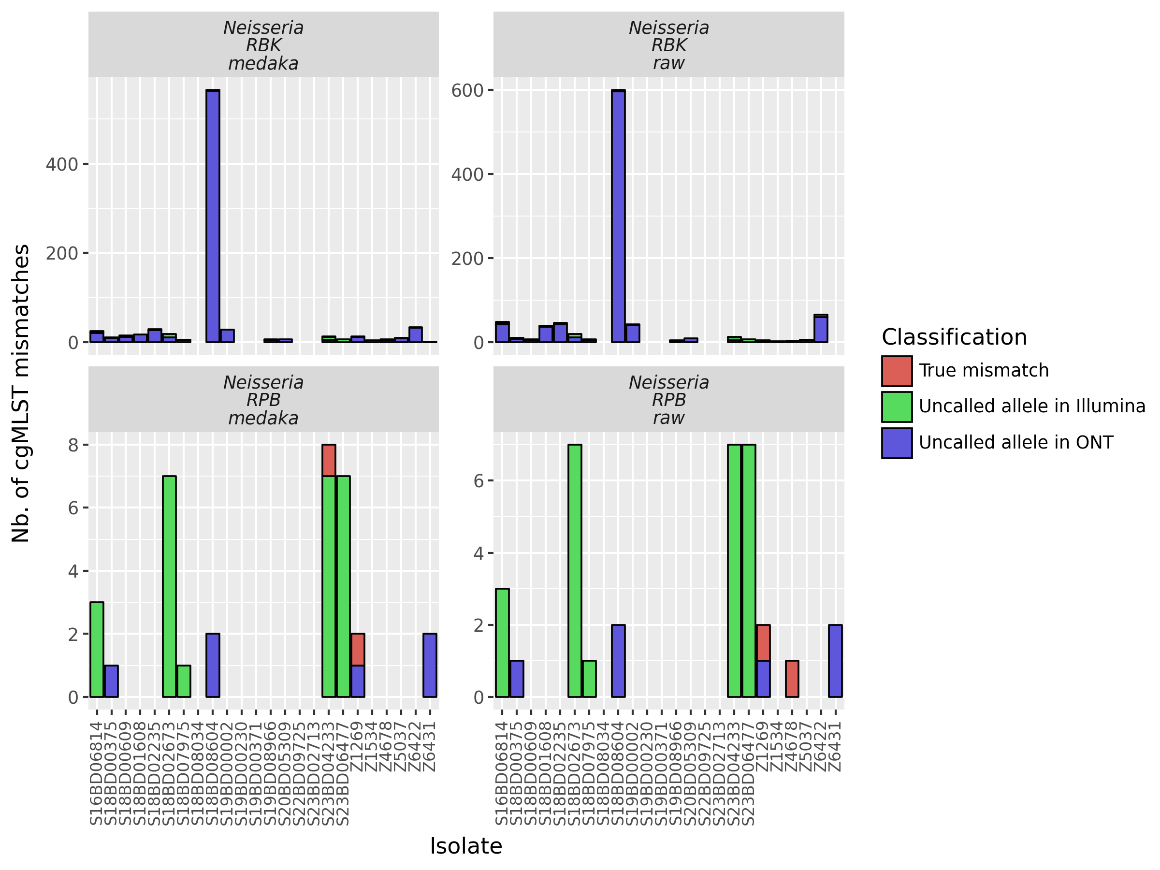
***

This plot shows the number of cgMLST loci mismatches between the ONT R10 assemblies and their corresponding Illumina-only assemblies. The x-axis represents individual isolates, while the y-axis shows the number of cgMLST mismatches between assemblies. The scale of the y-axis is different for each sub-plot. Mismatches are colored according to the mismatch classification given in the legend. Note that *Neisseria* sample S18BD02673 was of low quality, which may explain the high number of mismatches.

## **Figure S13: Mismatched cgMLST loci in the ONT-only datasets compared to Illumina for the *Salmonella* datasets**

***
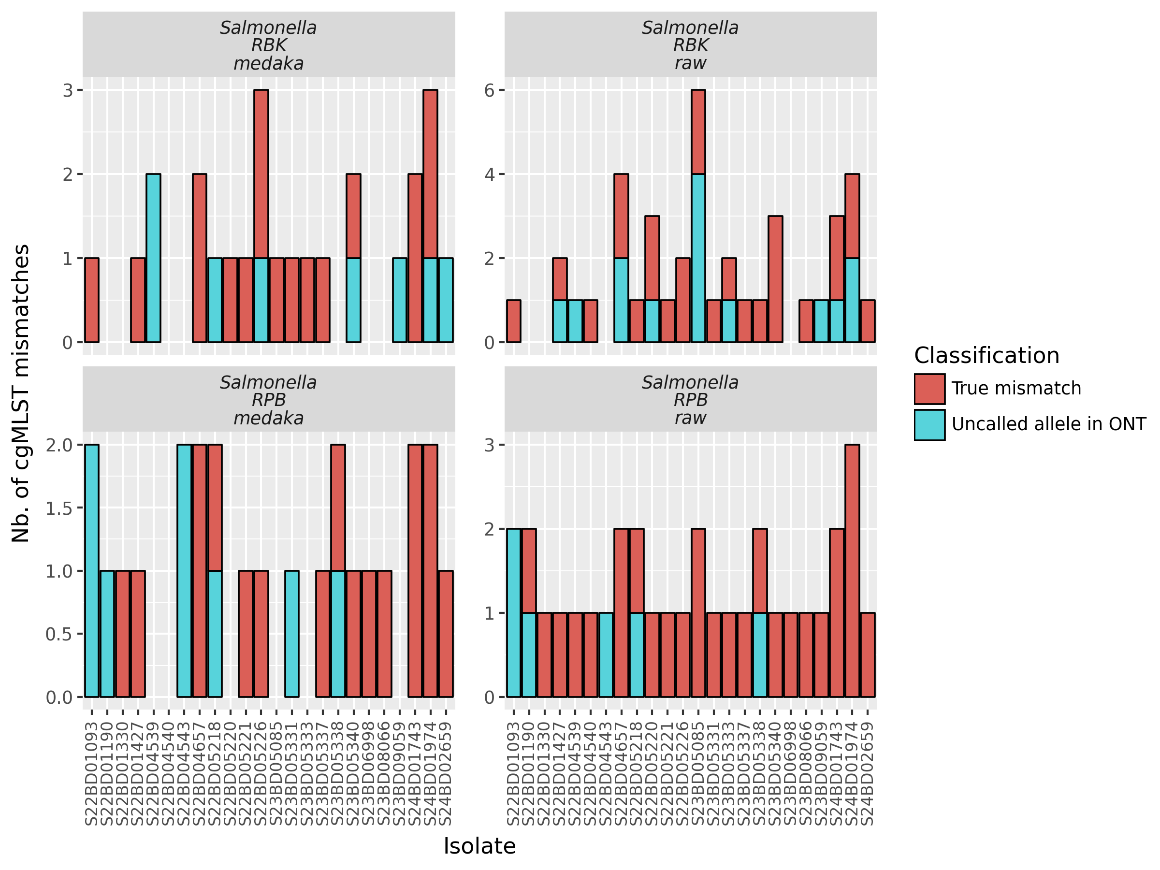
***

This plot shows the number of cgMLST loci mismatches between the ONT R10 assemblies and their corresponding Illumina-only assemblies. The x-axis represents individual isolates, while the y-axis shows the number of cgMLST mismatches between assemblies. The scale of the y-axis is different for each sub-plot.

## **Figure S14: SNPs and indels identified between the hybrid assembly and unpolished ONT-only assemblies in the cgMLST loci in the *Neisseria* datasets**


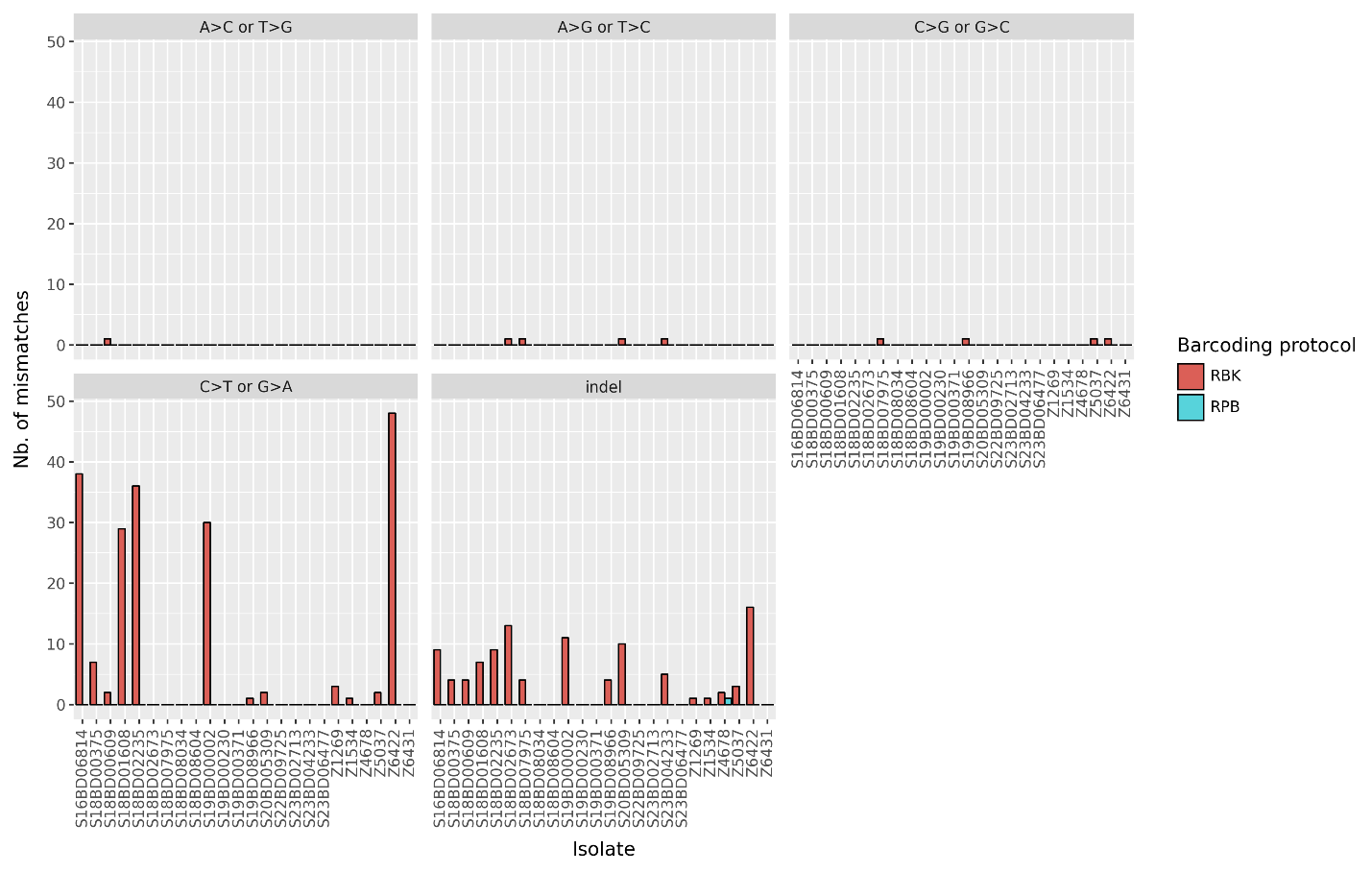


This figure shows the number of differences within the cgMLST loci between the hybrid assembly and the unpolished ONT-only assemblies for the *Neisseria* datasets. The bars are colored according to the barcoding protocol. The y-axis represents the number of differences between the hybrid assembly and the unpolished ONT-only assembly, grouped by mutation type as indicated in the subplot titles. Complementary mutations such as C to T and G to A (i.e., the same mutation on the opposite strand) were combined. The x-axis shows the isolate. The low quality data set S18BD08604 has been omitted from this figure due to the extremely high number of mismatches.

## **Figure S15: Methylation status of SNP and indel positions in unpolished ONT-only assemblies compared to hybrid assemblies**

1. *Neisseria*


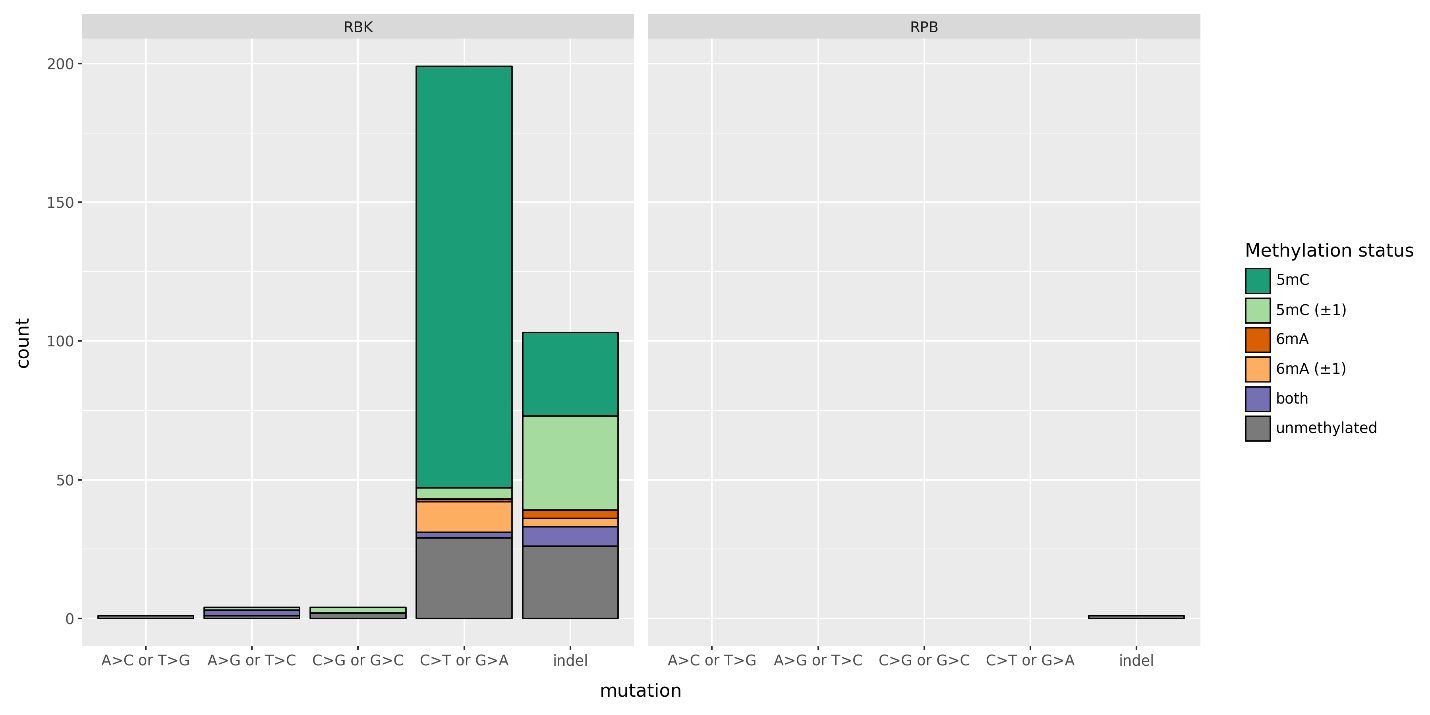


1. *Salmonella*


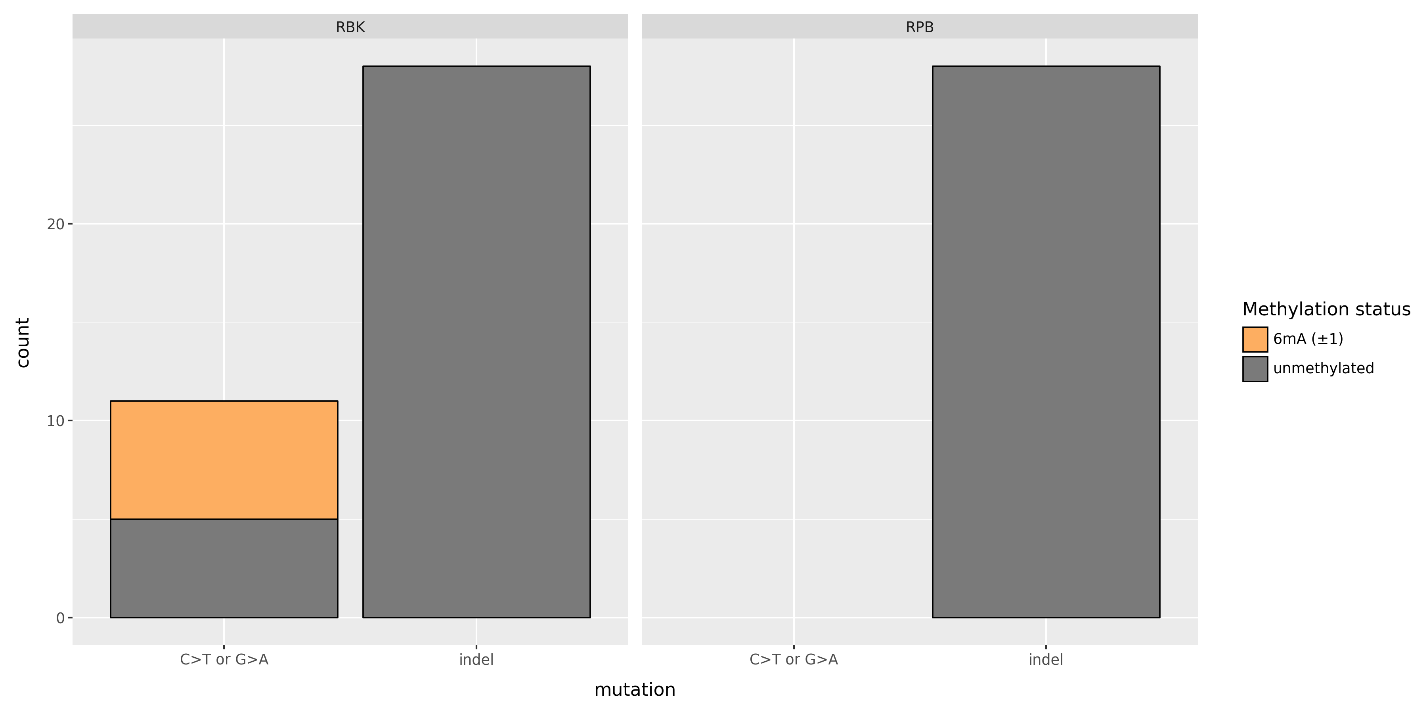


These figures show the methylation status for the discrepant positions within the cgMLST loci for the *Neisseria* (a) and *Salmonella* (b) datasets. The y-axis represents the number of mismatches across all datasets generated with the corresponding barcode. The x-axis represents the type of mismatch. The colors indicate the methylation status at the given position or its immediately adjacent positions. Adjacent positions are only considered if the given position itself was not called as methylated. The ‘both’ category corresponds to positions where both the upstream and downstream positions of the target position were reported as methylated.

## **Figure S16: Alignment at a methylated position resulting in a cgMLST mismatch**


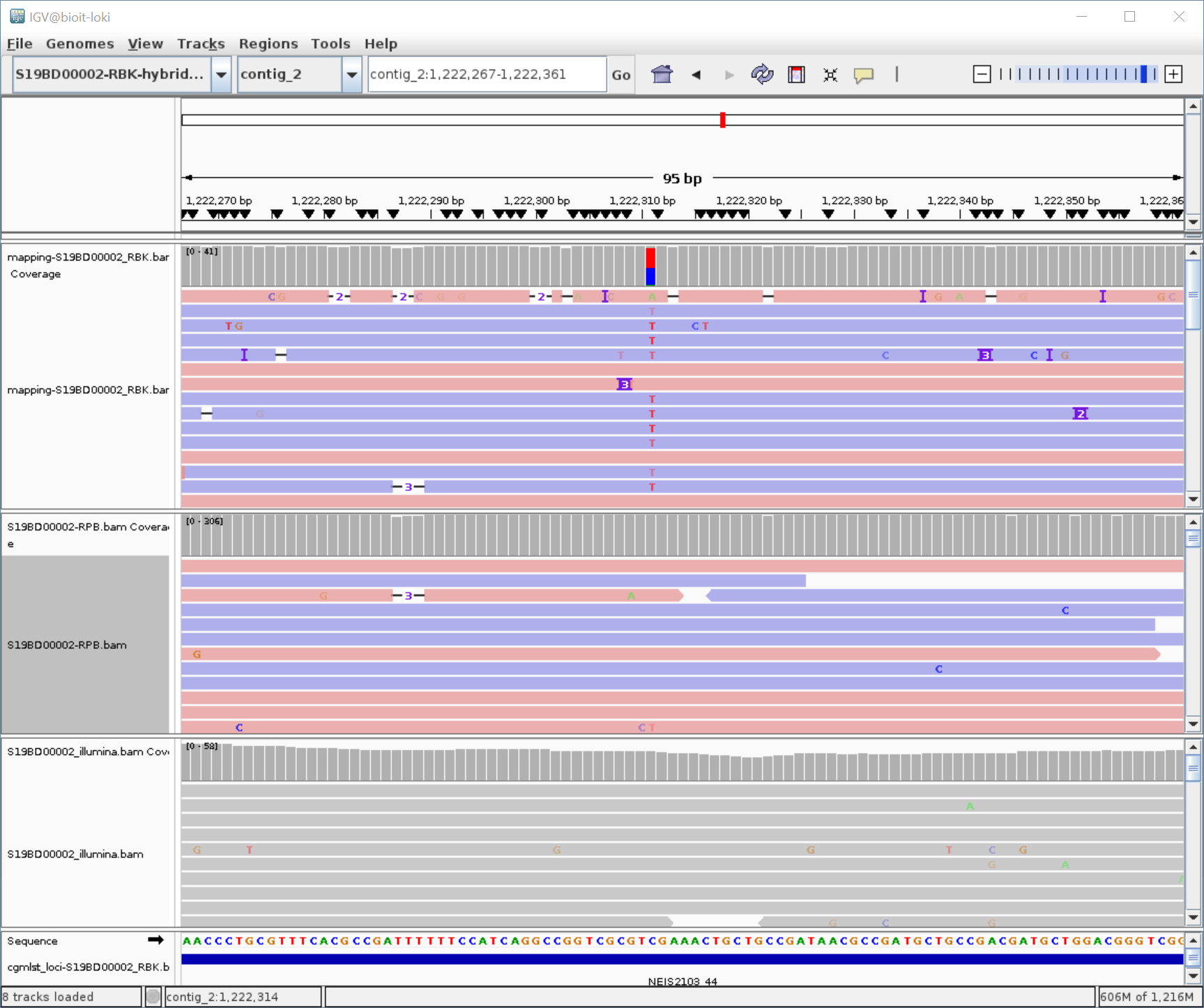


This visualization shows the mapping of the ONT RBK (top), ONT RPB (middle), and Illumina (bottom) reads for the S19BD00002 isolate to the NEIS2103_44 allele sequence in the hybrid assembly generated with the RBK data. Alignments are colored by strand orientation. At position 1,222,311 in the RBK hybrid assembly, identified as a 5mC methylated site, the RBK reads show an approximately equal mixture of T and C nucleotides, with a clear association to strand orientation. Depending on how this nucleotide is incorporated into the assembly (i.e., C or T), it may result in a mismatched in the NEIS2109 cgMLST locus. Note that this mixture of nucleotides is absent from the RPB and Illumina data. The visualization was generated using IGV v2.8.0 [59].

## **Figure S17: Homopolymer length for the indels in the cgMLST loci between hybrid and unpolished ONT-only datasets**


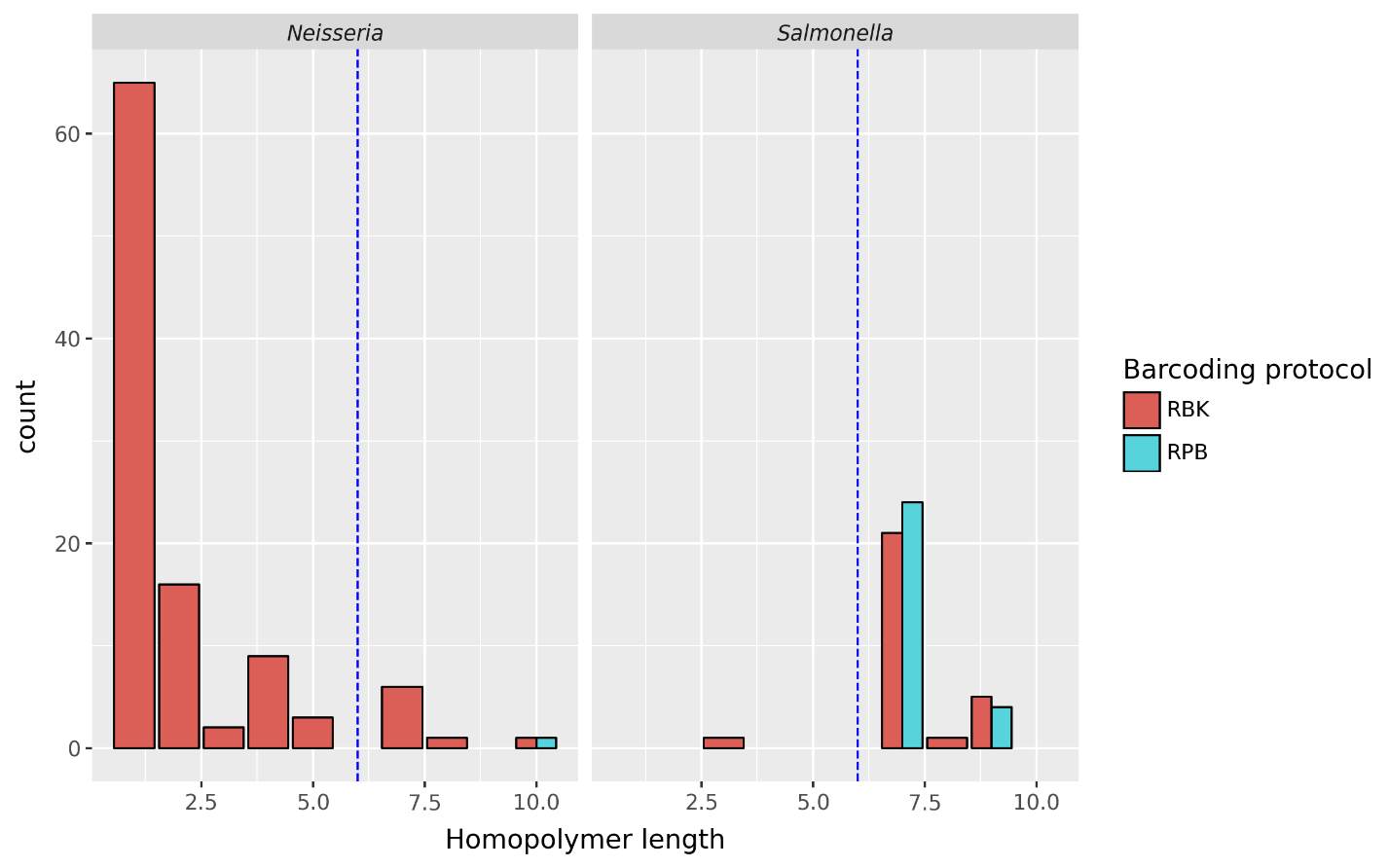


This figure shows the length of the homopolymers for the indel mismatches between the hybrid assemblies and corresponding unpolished ONT-only datasets. The y-axis represent the number of mismatches, the x-axis shows the homopolymer length. Bars are colored according to the barcoding protocol. The vertical blue line corresponds to the threshold of six bases to classify a kmer as a homopolymer.

## **Figure S18: SNPs and indels identified between the hybrid assembly and unpolished ONT-only assemblies in the cgMLST loci in the *Salmonella* datasets**


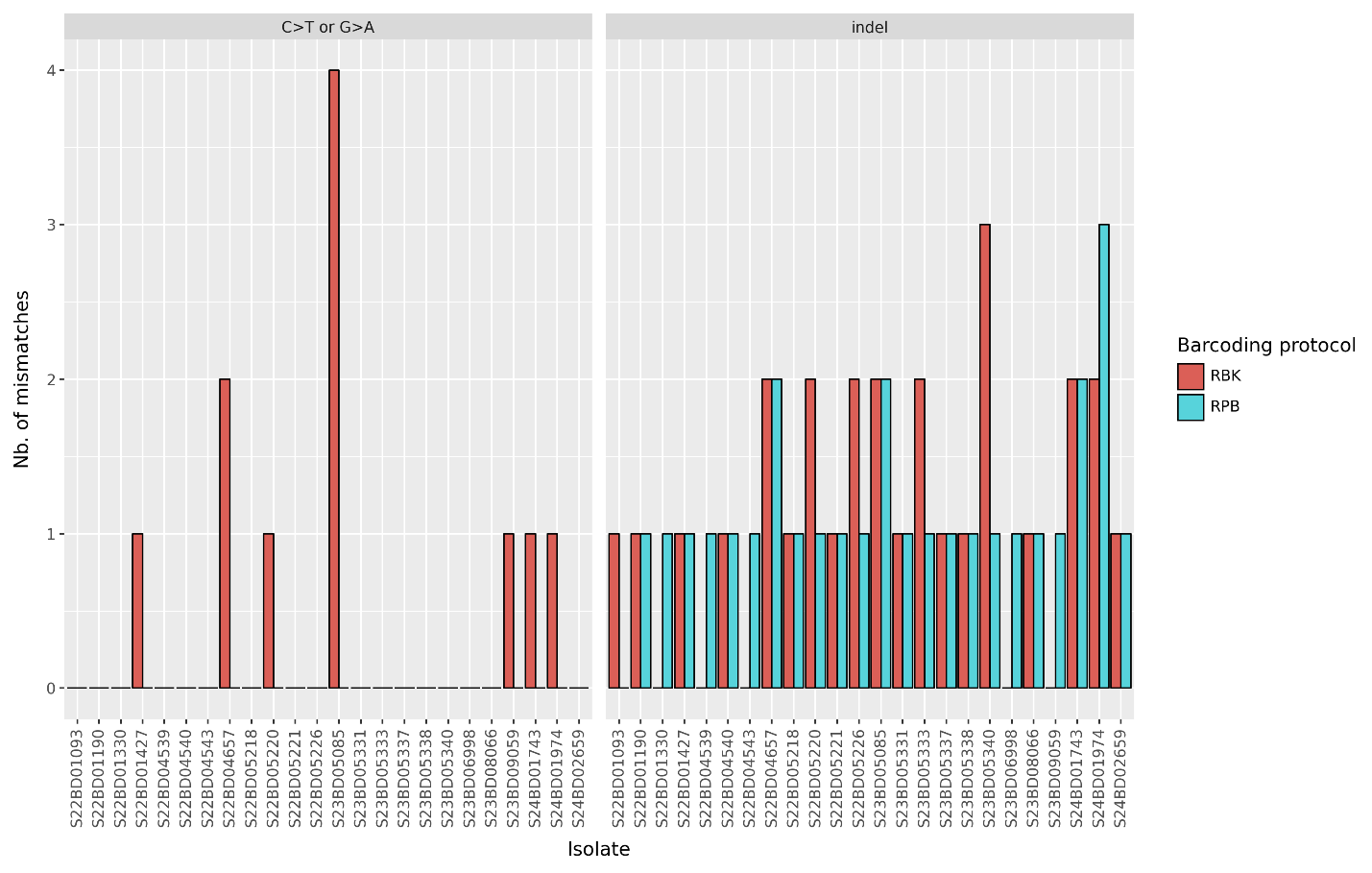


This figure shows the number of differences within the cgMLST loci between the hybrid assembly and the unpolished ONT-only assemblies for the *Salmonella* datasets. The bars are colored according to the barcoding protocol. The y-axis represents the number of differences between the hybrid assembly and the unpolished ONT-only assembly, grouped by mutation type as indicated in the subplot titles. Complementary mutations such as C to T and G to A (i.e., the same mutation on the opposite strand) were combined. The x-axis shows the isolate.

## **Figure S19: Visualization of a homopolymer error in the *Salmonella* S22BD01093 RBK dataset**


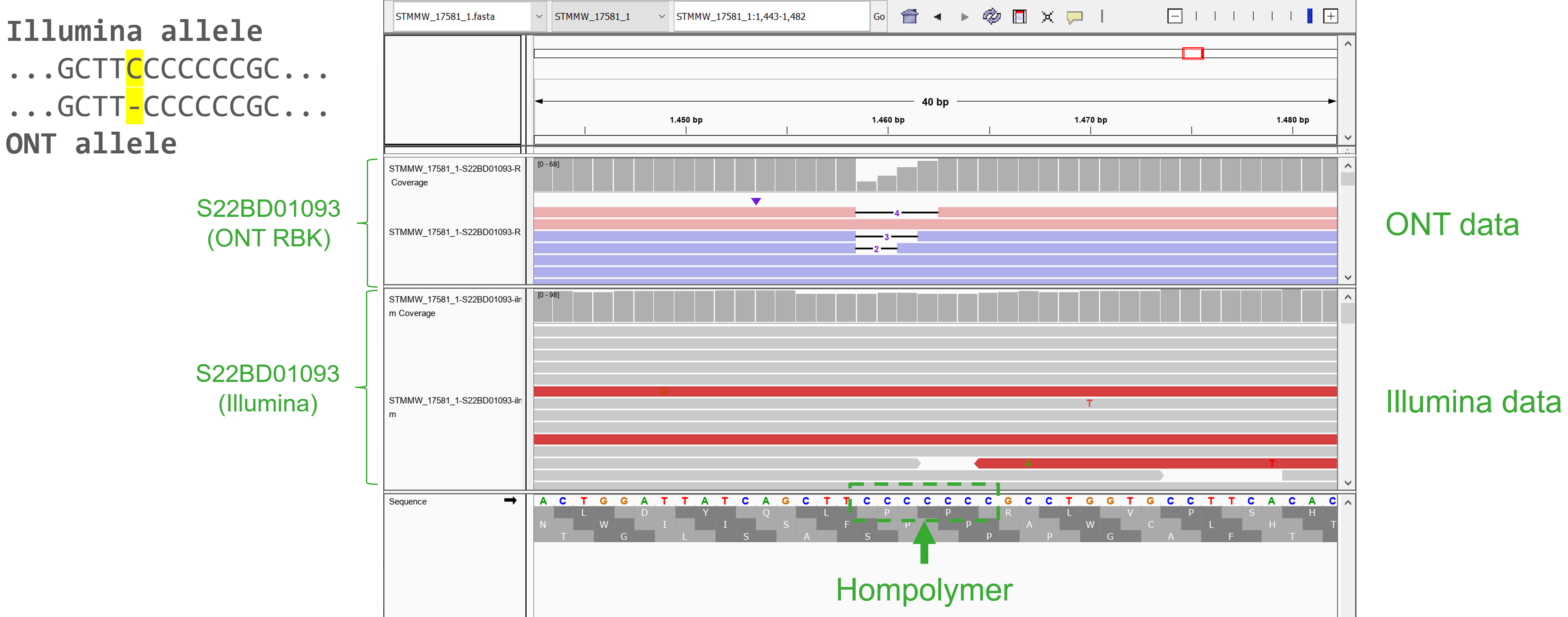


This visualization shows the mapping of the ONT RBK and Illumina reads for the S22BD01093 isolate to the STMMW_17581_1 allele sequence. The locus sequence is shown at the bottom with the ‘C’ homopolymer highlighted. In the ONT data, the number of C’s in the reads decreases as the length of the homopolymer increases, unlike in the Illumina data. Consequently, the consensus sequence obtained with ONT is one C shorter than expected, as illustrated in the top left corner. The visualization was generated using IGV v2.8.0 [59].

## **Figure S20: Minimum spanning tree for the *Neisseria* unpolished ONT RPB and *Salmonella* unpolished ONT RBK assemblies combined with the corresponding Illumina datasets**

| 1. ***Neisseria* RBK + Illumina** | 1. ***Salmonella* RPB + Illumina** |
| --- | --- |
| 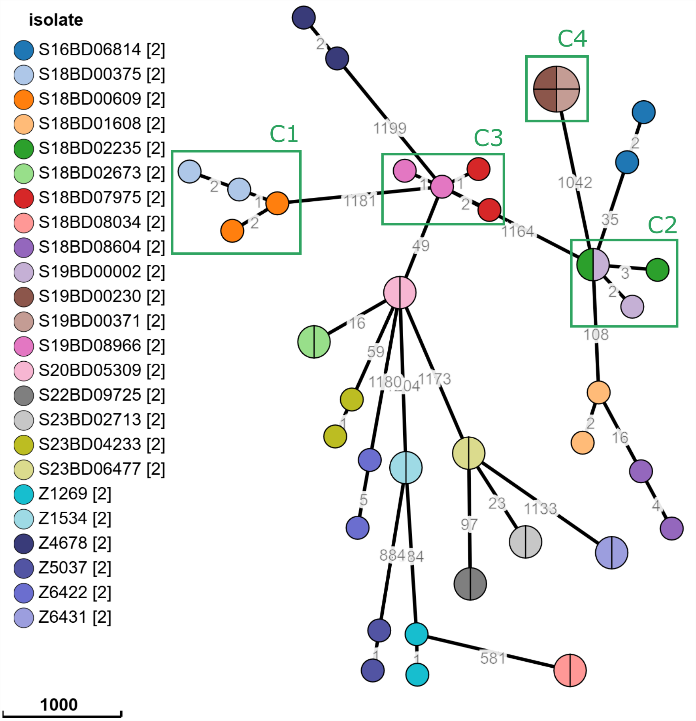 | 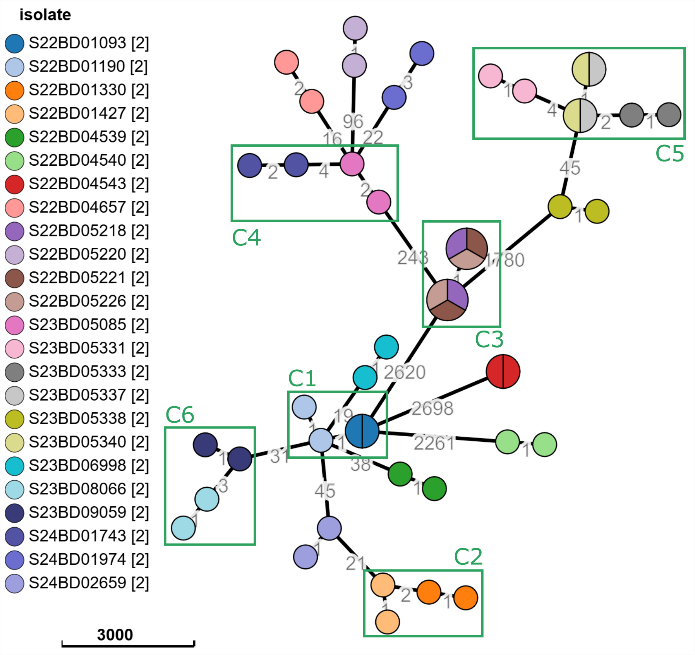 |

This plot shows the minimum spanning tree phylogeny combining (a) the ONT R10 RBK and the Illumina datasets as a network for the *Neisseria* isolates, and (b) the ONT R10 RPB and Illumina datasets for the *Salmonella* isolates. Branch lengths correspond to the number of cgMLST allele differences and are scaled logarithmically. Nodes are colored by isolate and clusters are indicated in green. A similar figure for the *Neisseria* RPB and *Salmonella* RBK datasets is provided in Figure 4.

# Tables

## **Table S1: ONT read statistics per run**

| **Run** | **Filter** | **Median read quality** | **Median read length** | **Nb. of reads** | **Nb. of bases** | **N50** |
| --- | --- | --- | --- | --- | --- | --- |
| *Neisseria* – RBK | No | 17.2 | 1,981 | 4,612,835 | 2.18E+10 | 11,301 |
| *Neisseria* – RPB | No | 19.4 | 4,126 | 3,523,664 | 1.51E+10 | 4,785 |
| *Salmonella* – RBK | No | 13.5 | 1,181 | 3,452,210 | 1.85E+10 | 17,315 |
| *Salmonella* – RPB | No | 16.4 | 3,761 | 4,740,134 | 1.91E+10 | 4,505 |
| *Neisseria* – RBK | Yes | 17.1 | 3,899 | 2,821,902 | 1.90E+10 | 11,447 |
| *Neisseria* – RPB | Yes | 18.0 | 4,187 | 3,236,422 | 1.42E+10 | 4,792 |
| *Salmonella* – RBK | Yes | 17.6 | 5,511 | 1,710,313 | 1.68E+10 | 17,988 |
| *Salmonella* – RPB | Yes | 17.8 | 3,833 | 4,313,672 | 1.78E+10 | 4,531 |

This table shows the read statistics for each of the four sequencing runs before and after filtering.

## **Table S2: Loci retained for the phylogenetic analysis**

| **Genus** | **Barcoding kit** | **Method** | **Loci after filtering (%)** |
| --- | --- | --- | --- |
| *Neisseria* | n/a | Illumina | 100.00 |
|  | RPB | Unpolished | 100.00 |
|  |  | Polished | 100.00 |
|  |  | Hybrid | 100.00 |
|  | RBK | Unpolished | 99.40 |
|  |  | Polished | 99.55 |
|  |  | Hybrid | 100.00 |
| *Salmonella* | n/a | Illumina | 96.90 |
|  | RPB | Unpolished | 96.90 |
|  |  | Polished | 96.90 |
|  |  | Hybrid | 96.90 |
|  | RBK | Unpolished | 96.90 |
|  |  | Polished | 96.87 |
|  |  | Hybrid | 96.90 |

This table shows the percentage of the cgMLST loci that were retained for the minimum spanning tree construction after filtering of the allele matrix, retaining only loci present in >75% of the included samples. The first column lists the genus, the second column lists the barcoding kit for the ONT data, the third column lists the assembly method, and the fourth column lists the percentage of loci retained. The *Neisseria* and *Salmonella* schemes contain 1329 and 3002 loci, respectively.

Abbreviations: not applicable (n/a).

## **Table S3: Percentage of cgMLST loci detected with the *Neisseria* cgMLST v1 scheme**

| **Sequencing technology & protocol** | **Assembly method** | **% cgMLST loci detected** |
| --- | --- | --- |
| RBK + Illumina | Hybrid | 98.35 |
| RPB + Illumina | Hybrid | 98.35 |
| RBK | ONT-only + medaka polishing | 97.69 |
| RPB | ONT-only + medaka polishing | 98.26 |
| RBK | ONT-only unpolished | 97.94 |
| RPB | ONT-only unpolished | 98.32 |
| Illumina | Illumina-only | 97.94 |

This table shows the median percentage of cgMLST loci detected for each sequencing technology and protocol, and assembly method using the first iteration of the *Neisseria* cgMLST scheme (1,605 loci). The main manuscript uses v3 of this scheme with 1329 loci. The percentage of cgMLST loci detected with v3 is provided in Table 2.

## **Table S4: Homopolymer errors outside of the cgMLST loci**

| **Genus** | **Kit** | **Indel errors** | | | |
| --- | --- | --- | --- | --- | --- |
|  |  | **Withing cgMLST loci** | | **Total** | |
|  |  | **In homopolymer** | **Not in homopolymer** | **In homopolymer** | **Not in homopolymer** |
| *Neisseria* | RBK | 15 | 1,212 | 243 | 3,005 |
|  | RPB | 1 | 0 | 164 | 337 |
| *Salmonella* | RBK | 27 | 12 | 108 | 152 |
|  | RPB | 28 | 0 | 112 | 525 |

This table lists the number of indel errors between the hybrid and corresponding ONT-only assemblies. Note that many of the indel errors in the RBK kit where associated with methylation (Supplementary Figure S15).

## **Table S5: BioSample accession numbers**

| **Species** | **Isolate** | **BioSample accession** | **Illumina sequencing platform** |
| --- | --- | --- | --- |
| *Neisseria meningitidis* | S16BD06814 | SAMN46909036 | MiSeq |
| *Neisseria meningitidis* | S18BD00375 | SAMN46909037 | MiSeq |
| *Neisseria meningitidis* | S18BD00609 | SAMN46909038 | MiSeq |
| *Neisseria meningitidis* | S18BD01608 | SAMN46909039 | MiSeq |
| *Neisseria meningitidis* | S18BD02235 | SAMN46909040 | MiSeq |
| *Neisseria meningitidis* | S18BD02673 | SAMN46909041 | MiSeq |
| *Neisseria meningitidis* | S18BD07975 | SAMN46909042 | MiSeq |
| *Neisseria meningitidis* | S18BD08034 | SAMN46909043 | MiSeq |
| *Neisseria meningitidis* | S18BD08604 | SAMN46909044 | MiSeq |
| *Neisseria meningitidis* | S19BD00002 | SAMN46909045 | MiSeq |
| *Neisseria meningitidis* | S19BD00230 | SAMN46909046 | MiSeq |
| *Neisseria meningitidis* | S19BD00371 | SAMN46909047 | MiSeq |
| *Neisseria meningitidis* | S19BD08966 | SAMN46909048 | MiSeq |
| *Neisseria meningitidis* | S20BD05309 | SAMN46909049 | MiSeq |
| *Neisseria meningitidis* | S22BD09725 | SAMN46909050 | MiSeq |
| *Neisseria meningitidis* | S23BD02713 | SAMN46909051 | MiSeq |
| *Neisseria meningitidis* | S23BD04233 | SAMN46909052 | MiSeq |
| *Neisseria meningitidis* | S23BD06477 | SAMN46909053 | MiSeq |
| *Neisseria meningitidis* | Z1269 | SAMN08874891 | MiSeq |
| *Neisseria meningitidis* | Z1534 | SAMN08874896 | MiSeq |
| *Neisseria meningitidis* | Z4678 | SAMN08874909 | MiSeq |
| *Neisseria meningitidis* | Z5037 | SAMN08874929 | MiSeq |
| *Neisseria meningitidis* | Z6422 | SAMN08874937 | MiSeq |
| *Neisseria meningitidis* | Z6431 | SAMN08874945 | MiSeq |
| *Salmonella enteritidis* | S22BD05218 | SAMN46909062 | MiSeq |
| *Salmonella enteritidis* | S22BD05220 | SAMN46909063 | MiSeq |
| *Salmonella enteritidis* | S22BD05221 | SAMN46909064 | MiSeq |
| *Salmonella enteritidis* | S22BD05226 | SAMN46909065 | MiSeq |
| *Salmonella enteritidis* | S23BD05085 | SAMN46909066 | NovaSeq |
| *Salmonella enteritidis* | S24BD01743 | SAMN46909075 | NovaSeq |
| *Salmonella enteritidis* | S24BD01974 | SAMN46909076 | NovaSeq |
| *Salmonella enteritidis* | S22BD04540 | SAMN46909059 | MiSeq |
| *Salmonella enteritidis* | S22BD01093 | SAMN46909054 | MiSeq |
| *Salmonella enteritidis* | S22BD01190 | SAMN46909055 | MiSeq |
| *Salmonella enteritidis* | S22BD01330 | SAMN46909056 | MiSeq |
| *Salmonella enteritidis* | S22BD01427 | SAMN46909057 | MiSeq |
| *Salmonella enteritidis* | S22BD04539 | SAMN46909058 | MiSeq |
| *Salmonella enteritidis* | S22BD04657 | SAMN46909061 | NovaSeq |
| *Salmonella enteritidis* | S23BD05331 | SAMN46909067 | NovaSeq |
| *Salmonella enteritidis* | S23BD05333 | SAMN46909068 | NovaSeq |
| *Salmonella enteritidis* | S23BD05337 | SAMN46909069 | NovaSeq |
| *Salmonella enteritidis* | S23BD05338 | SAMN46909070 | NovaSeq |
| *Salmonella enteritidis* | S23BD05340 | SAMN46909071 | NovaSeq |
| *Salmonella enteritidis* | S23BD06998 | SAMN46909072 | NovaSeq |
| *Salmonella enteritidis* | S23BD08066 | SAMN46909073 | NovaSeq |
| *Salmonella enteritidis* | S23BD09059 | SAMN46909074 | NovaSeq |
| *Salmonella enteritidis* | S24BD02659 | SAMN46909077 | NovaSeq |
| *Salmonella enteritidis* | S22BD04543 | SAMN46909060 | MiSeq |

Overview of the isolates used in this study. The columns list the genus, isolate name, NCBI accession number for the corresponding BioSample, and which Illumina sequencing platform was used for generation of short read data. All samples were also sequenced with both the ONT RPK and RBK protocols.

## **Table S6: cgMLST mismatches statistics for the ONT datasets**

| **Genus** | **Protocol** | **Method** | **Nb. of isolates** | **Mismatches (median)** | **Mismatches (minimum)** | **Mismatches (maximum)** |
| --- | --- | --- | --- | --- | --- | --- |
| *Neisseria* | RBK | ONT-only unpolished | 23 | 5 | 0 | 56 |
| *Neisseria* | RBK | ONT-only polished | 23 | 6 | 0 | 28 |
| *Neisseria* | RPB | ONT-only unpolished | 24 | 0 | 0 | 1 |
| *Neisseria* | RPB | ONT-only polished | 24 | 0 | 0 | 1 |
| *Salmonella* | RBK | ONT-only unpolished | 23 | 1 | 0 | 6 |
| *Salmonella* | RBK | ONT-only polished | 23 | 1 | 0 | 3 |
| *Salmonella* | RPB | ONT-only unpolished | 24 | 1 | 0 | 3 |
| *Salmonella* | RPB | ONT-only polished | 24 | 1 | 0 | 2 |

This plot shows the number of cgMLST loci mismatches between the hybrid assemblies and their corresponding ONT-only assemblies. The ONT data for the *Neisseria* S18BD08604 and *Salmonella* S23BD06998 isolates generated using the RBK kit were low-quality and these datasets were not included.

## **Table S7: Illumina read statistics**

| **Genus** | **Isolate** | **Estimated cov.** | **Read pairs (total)** | **Read pairs (after downsampling)** | **Read pairs (after trimming)** |
| --- | --- | --- | --- | --- | --- |
| *Neisseria* | S16BD06814 | 95 | 377,686 | 377,686 | 346,658 |
| *Neisseria* | S18BD00375 | 43 | 188,467 | 188,467 | 172,530 |
| *Neisseria* | S18BD00609 | 34 | 148,770 | 148,770 | 135,796 |
| *Neisseria* | S18BD01608 | 47 | 204,902 | 204,902 | 181,865 |
| *Neisseria* | S18BD02235 | 73 | 316,072 | 316,072 | 266,907 |
| *Neisseria* | S18BD02673 | 24 | 104,700 | 104,700 | 90,985 |
| *Neisseria* | S18BD07975 | 49 | 213,825 | 213,825 | 193,709 |
| *Neisseria* | S18BD08034 | 67 | 294,108 | 294,108 | 271,242 |
| *Neisseria* | S18BD08604 | 83 | 364,120 | 364,120 | 339,815 |
| *Neisseria* | S19BD00002 | 68 | 297,469 | 297,469 | 261,837 |
| *Neisseria* | S19BD00230 | 116 | 531,494 | 457,783 | 422,427 |
| *Neisseria* | S19BD00371 | 109 | 481,129 | 441,858 | 388,791 |
| *Neisseria* | S19BD08966 | 78 | 349,633 | 349,633 | 321,389 |
| *Neisseria* | S20BD05309 | 63 | 283,388 | 283,388 | 258,497 |
| *Neisseria* | S22BD09725 | 98 | 438,247 | 438,247 | 399,432 |
| *Neisseria* | S23BD02713 | 126 | 555,501 | 442,022 | 394,700 |
| *Neisseria* | S23BD04233 | 81 | 370,366 | 370,366 | 341,189 |
| *Neisseria* | S23BD06477 | 70 | 312,745 | 312,745 | 278,017 |
| *Neisseria* | Z1269 | 82 | 303,275 | 303,275 | 271,237 |
| *Neisseria* | Z1534 | 91 | 334,125 | 334,125 | 297,069 |
| *Neisseria* | Z4678 | 93 | 343,416 | 343,416 | 315,117 |
| *Neisseria* | Z5037 | 86 | 314,534 | 314,534 | 277,924 |
| *Neisseria* | Z6422 | 105 | 389,073 | 369,095 | 337,729 |
| *Neisseria* | Z6431 | 112 | 410,489 | 367,830 | 332,643 |
| *Salmonella* | S22BD01093 | 105 | 1,793,073 | 1,714,957 | 1,565,168 |
| *Salmonella* | S22BD01190 | 77 | 791,794 | 791,794 | 705,750 |
| *Salmonella* | S22BD01330 | 164 | 2,800,231 | 1,709,583 | 1,563,516 |
| *Salmonella* | S22BD01427 | 97 | 1,662,129 | 1,662,129 | 1,499,968 |
| *Salmonella* | S22BD04539 | 51 | 523,062 | 523,062 | 483,448 |
| *Salmonella* | S22BD04540 | 42 | 429,441 | 429,441 | 368,627 |
| *Salmonella* | S22BD04543 | 54 | 556,098 | 556,098 | 513,649 |
| *Salmonella* | S22BD04657 | 317 | 5,285,443 | 1,664,923 | 1,556,817 |
| *Salmonella* | S22BD05218 | 53 | 546,802 | 546,802 | 491,612 |
| *Salmonella* | S22BD05220 | 60 | 630,769 | 630,769 | 587,831 |
| *Salmonella* | S22BD05221 | 58 | 604,053 | 604,053 | 556,232 |
| *Salmonella* | S22BD05226 | 55 | 572,698 | 572,698 | 518,301 |
| *Salmonella* | S23BD05085 | 311 | 5,174,106 | 1,664,914 | 1,546,796 |
| *Salmonella* | S23BD05331 | 315 | 5,244,050 | 1,665,030 | 1,545,961 |
| *Salmonella* | S23BD05333 | 557 | 9,279,144 | 1,665,852 | 1,562,117 |
| *Salmonella* | S23BD05337 | 692 | 11,511,909 | 1,666,110 | 1,561,266 |
| *Salmonella* | S23BD05338 | 571 | 9,502,586 | 1,666,020 | 1,559,747 |
| *Salmonella* | S23BD05340 | 403 | 6,713,741 | 1,665,014 | 1,562,331 |
| *Salmonella* | S23BD06998 | 622 | 10,348,203 | 1,665,875 | 1,541,499 |
| *Salmonella* | S23BD08066 | 900 | 14,984,610 | 1,665,453 | 1,617,053 |
| *Salmonella* | S23BD09059 | 400 | 6,651,234 | 1,665,264 | 1,590,420 |
| *Salmonella* | S24BD01743 | 523 | 8,817,392 | 1,687,055 | 1,579,976 |
| *Salmonella* | S24BD01974 | 436 | 7,327,839 | 1,679,667 | 1,567,509 |
| *Salmonella* | S24BD02659 | 261 | 4,448,077 | 1,700,371 | 1,573,999 |

This table lists the number of read pairs for the Illumina datasets. The first and second columns list the genus and isolate name, respectively. The third column contains the estimated coverage based on the number of bases and the expected genome size. The fourth, fifth and sixth columns list the number of input read pairs, read pairs after downsampling (for datasets with estimated coverage >100x), and the read pairs after trimming.

# Methods

## **Whole-genome sequencing (Illumina)**

The Illumina datasets were generated using the Illumina MiSeq and the Illumina NovaSeq instruments, as indicated in Table S5. For the Illumina MiSeq, short-read DNA libraries were prepared using the Nextera XT DNA library preparation kit (Illumina, San Diego, CA, USA) according to the manufacturer’s instructions. Sequencing was performed on an Illumina MiSeq sequencer using the V3 chemistry, obtaining 250 bp paired-end reads, aiming for a theoretical coverage of 60x per isolate based on the expected genome sizes of ~2.2 Mbp and ~5 Mbp for *Neisseria* and *Salmonella*, respectively*.* Illumina NovaSeq sequencing was performed at Eurofins (Germany), using high-throughput sequencing TruSeq DNA library preparation and NovaSeq PE 150 sequence mode, aiming for a minimal genome coverage of 100x.

## **Data preprocessing, filtering and *de novo* assembly of the Illumina data**

The Illumina datasets were generated using the Illumina MiSeq and the Illumina NovaSeq instruments, as indicated in Table S5. For the Illumina MiSeq, short-read DNA libraries were prepared using the Nextera XT DNA library preparation kit (Illumina, San Diego, CA, USA) according to the manufacturer’s instructions. Sequencing was performed on an Illumina MiSeq sequencer using the V3 chemistry, obtaining 250 bp paired-end reads, aiming for a theoretical coverage of 60x per isolate based on the expected genome sizes of ~2.2 Mbp and ~5 Mbp for *Neisseria* and *Salmonella*, respectively*.* Illumina NovaSeq sequencing was performed at Eurofins (Germany), using high-throughput sequencing TruSeq DNA library preparation and NovaSeq PE 150 sequence mode, aiming for a minimal genome coverage of 100x.

# Results

## **WGS data quality and yield (Illumina data)**

The assembly metrics for the Illumina-only assemblies are shown in Supplementary Figure S5. Note that datasets with estimated coverage >100x were downsampled to ~100x before *de novo* assembly. The estimated coverage and number of read pairs before and after trimming are provided in Supplementary Table S7. After downsampling, the median coverage for the *Neisseria* and *Salmonella* datasets were 58.5x and 90.5x, respectively. All Illumina datasets were above the 20x sequencing depth threshold enforced in our in-house workflows [25], [46], except for the *Neisseria* S18BD02673 sample, which had a median coverage of 19x. Notwithstanding, since 99.47% of cgMLST loci could be identified (Section 3.2), this sample was retained for further analysis. The Illumina-only assemblies were much more fragmented than the corresponding ONT assemblies, indicated by lower N50 values and a higher number of contigs. For *Neisseria*, the median N50 of the Illumina assemblies was 49,506 bp, compared to 818,460 bp and 2,193,100 bp for the ONT RPB and RBK kits. Similarly, for *Salmonella*, the median N50 was 284,909 bp compared to 1,532,551 bp and 4,831,397 bp for the RPB and RBK kits, respectively. The median length of the largest contig for the Illumina assemblies was 108,438 bp for *Neisseria* and 386,747 bp for *Salmonella*. In contrast to the ONT datasets, circular contigs covering complete chromosomes could not be obtained for any of the isolates. The total assembly length was within the expected range for all datasets. To summarize, the quality of all Illumina datasets was sufficient for subsequent analysis.
